# Time-Course Transcriptome, Metabolome, and Weighted Gene Co-Expression Network Analysis Reveal the Roles of the *OsBELH4A* Gene in Regulating Leaf Senescence and Grain Yield of Rice

**DOI:** 10.3390/plants14192973

**Published:** 2025-09-25

**Authors:** Ruyi Zheng, Tianyu Chen, Jianjian Li, Chengcheng Hu, Zhiming Yu, Zhanghui Zeng, Zhehao Chen, Lilin Wang, Taihe Xiang, Xiaoping Huang

**Affiliations:** College of Life and Environmental Sciences, Hangzhou Normal University, Hangzhou 311121, China; 2023210304063@stu.hznu.edu.cn (R.Z.); 2024111010022@stu.hznu.edu.cn (T.C.); 2021111023026@stu.hznu.edu.cn (J.L.); 2023111010040@stu.hznu.edu.cn (C.H.); yuzhiming@hznu.edu.cn (Z.Y.); zhzeng@hznu.edu.cn (Z.Z.); zhchen@hznu.edu.cn (Z.C.); llwang@hznu.edu.cn (L.W.); xthcn@hznu.edu.cn (T.X.)

**Keywords:** grain yield, metabolome, *OsBELH4A*, premature senescence, transcriptome, WGCNA

## Abstract

Rice (*Oryza sativa* L.) is one of the major food crops. Yield and quality are affected by premature leaf senescence, a complex and tightly regulated developmental process. To elucidate the molecular regulatory mechanism controlling rice leaf senescence, the integrative transcriptome, metabolome and weighted gene co-expression network analysis (WGCNA) of flag leaves in five development stages (FL1–FL5) was performed. In this study, a total of 9412 differential expressed genes (DEGs) were identified. To further mine DEGs related to leaf senescence, a total of five stage-specific modules were characterized by WGCNA. Among them, two modules displayed continuous down-regulated and up-regulated trends from stages FL1 to FL5, which were considered to be highly negatively and positively correlated with the senescence trait, respectively. GO enrichment results showed that the genes clustered in stage-specific modules were significantly enriched in a vast number of senescence-associated biological processes. Furthermore, large numbers of senescence-related genes were identified, mainly participating in transcription regulation, hormone pathways, degradation of chlorophyll, ROS metabolism, senescence-associated genes (SAGs), and others. Most importantly, a total of 40 hub genes associated with leaf senescence were identified. In addition, the metabolome analysis showed that a total of 309 differential metabolites (DMs) were identified by WGCNA. The integrative transcriptome and metabolome analysis identified a key hub gene *OsBELH4A* based on the correlation analysis conducted between 40 hub genes and 309 DMs. The results of function validation showed that *OsBELH4A* overexpression lines displayed delayed leaf senescence, and significantly increased grain number per plant and grain number per panicle. By contrast, its knockout lines displayed premature leaf senescence and reduced grain yield. Exogenous hormone treatment showed that *OsBELH4A* significantly responded to SA and auxin. These findings provide novel insights into leaf senescence, and further contribute to providing genetic resources for the breeding of crops resistant to premature senescence.

## 1. Introduction

Senescence is a natural phenomenon in the plant kingdom and constitutes the final stage of leaf development, which involves the process of programmed cell death (PCD) [1]. During the senescence period, leaves undergo dramatically coordinated changes at the physiological, biochemical, and molecular levels. For example, chlorophyll degradation and chloroplast decomposition cause leaf yellowing. The biomacromolecules including carbohydrates, lipids, proteins, and nucleic acids are hydrolyzed. In addition, the nutrients are allocated to other organs or tissues, such as young leaves or developing seeds [1]. Leaf senescence is orchestrated by a complex interplay of environmental signals and endogenous cues. The former mainly includes salt stress, extreme temperatures, light conditions, high sugar content and pathogens [2]. The latter mainly includes hormones, leaf age and foliar reactive oxygen species (ROS) levels; for instance, ethylene, jasmonic acid, salicylic acid, abscisic acid, brassinosteroids and strigolactones, which can accelerate leaf senescence, while cytokinins, gibberellins, and auxins delay leaf senescence [3]. Rice (*Oryza sativa* L.) is one of the world’s most important food crops. Premature leaf senescence will shorten the functional period of leaves, reduce the assimilation and transportation of substances, accelerate the grain-filling rate, and ultimately affect rice yield and quality [4]. Furthermore, some studies have reported that for every one-day delay in the senescence of rice leaves during the grain-filling period, the rice yield can theoretically increase by 2% and practically increase by 1% [5,6]. Therefore, the appropriate timing of leaf senescence onset and progression is crucial for crop yield and quality during the crop plant’s life cycle.

At the molecular level, the senescence syndrome is reflected by the changes in the expression levels of senescence-associated genes (SAGs). SAGs are up-regulated genes and can be subdivided into two sub-categories. For example, senescence-specific genes are only activated during the senescence process and show an increased expression level. It has been reported that the expression level of *OsSAG12-2* in dark-induced senescent leaves is significantly higher than that in the control plants [7]. The other category of genes shows low expression in the early growth stage of leaves and high expression after the onset of senescence [8]. More importantly, researchers have map-cloned a larger number of SAGs, which are functionally validated to play vital roles in leaf senescence. In detail, these SAGs are classified into the following: (I) transcription factors *OsNAP* [9], *OsNAC103* [10], *OsWRKY53* [11], and *OsWRKY93* [12]; (II) chlorophyll-degradation-related genes *NYC1* [13], *NOL* [14], *Lhca4* [15], and *SGR* [16]; (III) hormone-related genes *OsMTS1* [17], *OsCOMT* [18], and *OsCKX11* [19]; (IV) ROS-related genes *OsWSS1* [20], *HPA1* [21], *PWL1* [22], and *OsLHT1* [23]; (V) other genes *RAmy1A* [24] and *OsSWEET1b* [25].

Furthermore, an increasing number of studies have been performed to mine and validate the gene function in the development process of plants based on the integrative transcriptome, metabolome, and weighted gene co-expression network analysis (WGCNA). For example, the findings indicated that the greater accumulation of a spectrum of metabolites, particularly sphingosine and chlorogenic acid, promoted cold tolerance in cold-tolerant citrus species [26]. Based on integrated omics including anthocyanin and the flavonoid metabolome and transcriptome, the substances’ basis and metabolic mechanisms regulating gradient coloration were thoroughly elucidated. In addition, the novel transcription factors *VvbHLH148*, *VvMYBA22*, and *VvMYBA3* were also most probably involved in this regulatory process [27]. Transcriptome and weighted gene co-expression network analysis identified transcription factor *MsMYB12*, which can directly bind to the promoter of *MsFLS13*. Further results showed that *MsFLS13* overexpression enhanced flavonol accumulation and antioxidant capacity, which improved combined stress tolerance [28]. WGCNA uncovered a key gene alpha-globulin1 (GLB1) in co-expression networks, and the loss of GLB1 function caused significant changes in seed storage proteins, reduced amylose content, altered starch granules, and modified pasting properties without affecting plant phenotypes [29]. To date, no study has mined the underlying regulatory genes for rice leaf senescence based on transcriptome, metabolome, and WGCNA.

BELL/BLH (BEL1-like homeodomain) transcription factors belong to a sub-family of the TALE superfamily. They have a three-amino-acid loop extension and a conserved “PYP” (proline–tyrosine–proline) sequence in the homeodomain [30]. BLH transcription factors have different functions depending on the species and target genes [31]. In *Arabidopsis thaliana*, the BELL/BLH transcription factor family consists of 13 members. Among them, *BEL1* was involved in ovule development, and *BLH9* and *BLH8* regulated plant bud structure [32]. In the tomato, BELL/BLH transcription factor SlBEL2 can interact with the SlGLK2 protein to inhibit the transcriptional activity of its downstream target genes, thereby affecting chloroplast development and suppressing the formation of green shoulders [33]. In cotton, BELL/BLH transcription factor *GhBLH1* activated the transcription of *GhFAD7A-1*, which in turn enhanced the accumulation of linolenic acid and promoted the elongation of cotton fiber cells [34]. In *Lactuca sativa*, a BEL1 transcription factor *LsSAW1* promoted the formation of multiple-leaf heads by down-regulating the adaxial leaf gene *LsAS1* and up-regulating the abaxial leaf gene *LsYAB1* [35]. These studies demonstrated the diverse roles of BELL/BLH transcription factors in the plant growth and development process. In this study, we identified a key BELL/BLH (BEL1-like homeodomain) transcription factor *OsBELH4A* based on the multi-omics and WGCNA method. However, the biological function is not clear.

To explore the function of *OsBELH4A* in rice, the overexpression and CRISPR/Cas9 vectors were constructed followed by genetic transformation. The results showed that the *OsBELH4A* gene negatively regulated leaf senescence and had potential impacts on rice yield.

## 2. Results

### 2.1. Senescence Feature, DEG Analysis and Weighted Gene Co-Expression Network Analysis

**Figure 1:**
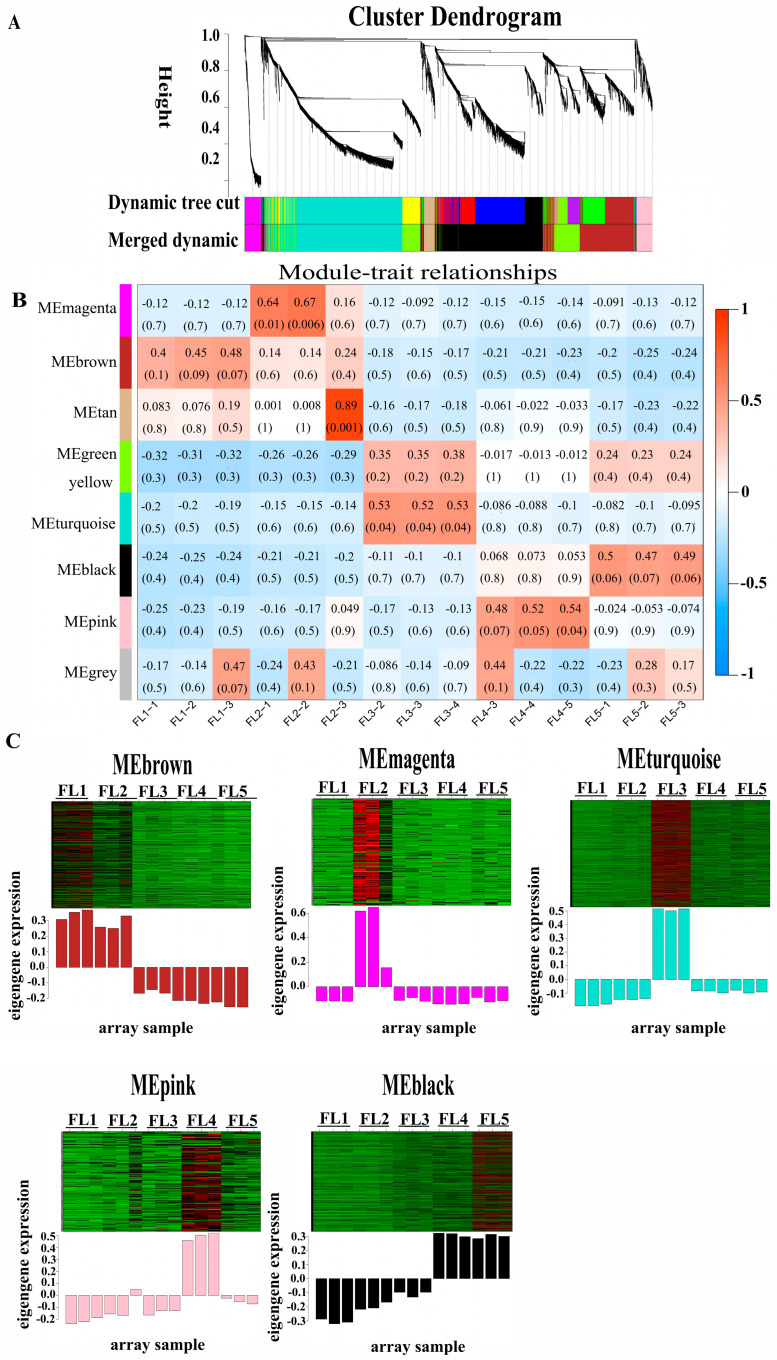
Weighted gene co-expression network analysis (WGCNA) of differential expressed genes (DEGs) in flag leaves of rice sampled at booting stage, flowering stage, early-senescence stage, mid-senescence stage, and late-senescence stage. (**A**) Hierarchical cluster tree (cluster dendrogram) indicating eight modules of co-expressed genes. Each leaf in the tree indicates each of the DEGs, and major tree branches indicate each of the modules designated as various colors. (**B**) Heatmap of modules–trait relationships. Each row represents a module (refers to a cluster of genes that show highly correlated expression patterns across samples, which are often labeled with arbitrary colors for easy reference), and each column represents a developmental stage. The intersection cells display correlation coefficients and *p*-values (inside parentheses) between the modules and stages. Red cells indicate strong positive correlations between a specific module and a developmental stage, whereas blue cells indicate strong negative correlations. (**C**) Heatmap of the five different module genes, with red indicating high expression, and green indicating low expression. The upper part is the heat map of gene expression within the modules, and the lower part is the bar plot of the expression of module eigengenes in each sample. Eigengene was considered as a representative of the gene expression profiles in a module.

**Figure 2:**
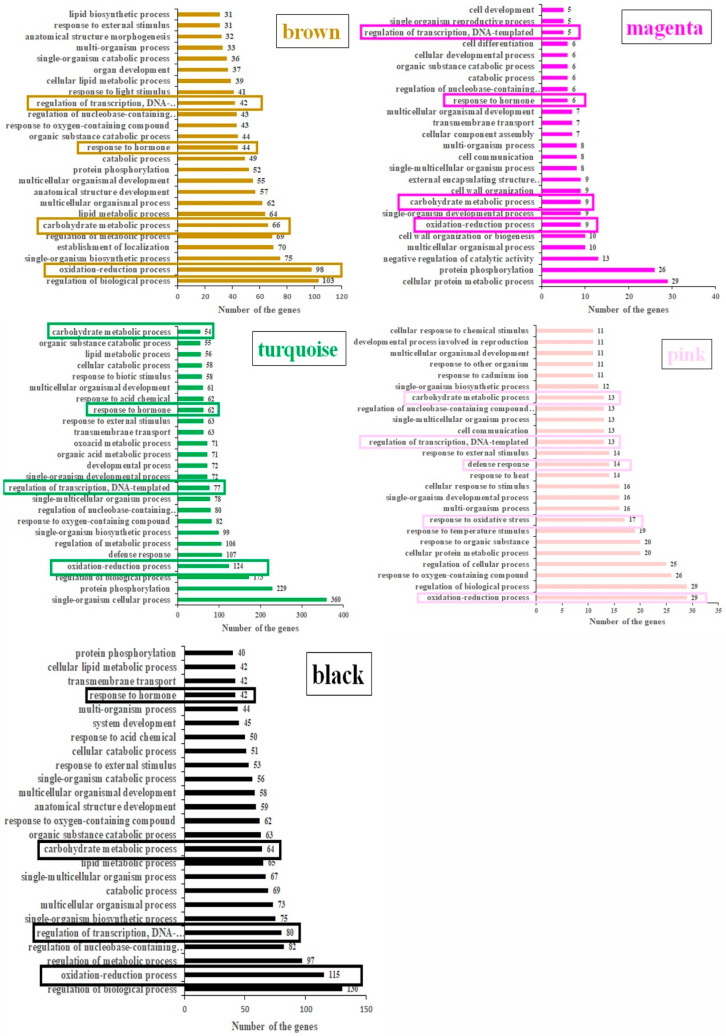
GO enrichment analysis of DEGs in the five stage-specific modules. The value on the horizontal axis represents the number of genes participating in related biological processes in each module. The biological processes that are boxed with rectangles indicate they are enriched in all modules.

Prior to sequencing, the chlorophyll content of the flag leaves sampled at booting stage (FL1, 9 days before flowering), flowering stage (FL2, 3 days after flowering), early-senescence stage (FL3, 9 days after flowering), mid-senescence stage (FL4, 19 days after flowering), and late-senescence stage (FL5, 29 days after flowering) were measured (Supplementary Figure S1A,B). The results showed that the chlorophyll content of sample FL3 significantly decreased (Supplementary Figure S1C). Furthermore, the expression levels of marker genes (*SGR* and *OsNAP*) were significantly increased from FL3 stage (Supplementary Figure S1D,E). These findings indicated the initiation of leaf senescence at FL3 stage. After sequencing, a total of 9412 DEGs were identified and annotated based on the screening criteria of log2 (fold change) > 1 and false discovery rate < 0.05 (Supplementary Table S1). To identify the gene expression regulatory network related to leaf senescence in rice, a WGCNA was performed. The dynamic tree-cutting algorithm generated a dendrogram of clustered modules, grouping the co-expressed genes into eight modules (Figure 1A). Based on the correlation coefficients between module eigengenes and different stages (FL1–FL5), a total of five stage-specific modules (MEbrown, MEmagenta, MEturquoise, MEpink, and MEblack) were obtained (Figure 1B, labeled with arbitrary colors for easy reference). Heatmaps indicating the gene expression profiles of different module genes in array samples (FL1–FL5) at different stages were visualized (Figure 1C). The MEbrown, MEmagenta, MEturquoise, MEpink, and MEblack modules, respectively, comprised 801, 191, 1533, 222, and 1088 DEGs, which separately displayed the highest expression level at FL1, FL2, FL3, FL4, and FL5 (Figure 1C; Supplementary Table S2). To explore the functional characteristics of DEGs in a stage-specific module, the GO enrichment and KEGG enrichment analysis were performed. The results showed that the DEGs in five modules were significantly enriched in the biological process, including the oxidation reduction process (GO:0055114), carbohydrate metabolic process (GO:0005975), response to hormone (GO:0009725), and regulation of transcription, DNA-templated (GO:0006355), except for the defense response (GO:0006952) that was specifically activated in the FL4 stage (Figure 2; Supplementary Table S3). Furthermore, the KEGG enrichment showed that DEGs in the five modules were mainly involved in pathways related to leaf senescence including photosynthesis–antenna proteins (ko00196), tryptophan metabolism (ko00380), phenylalanine metabolism (ko00360), alpha-Linolenic acid metabolism (ko00592), and plant hormone signal transduction (ko04075) (Supplementary Figure S2; Supplementary Table S4).

### 2.2. Identification of Transcription Factors in Five Stage-Specific Modules

It has been reported that transcription factors played essential roles in regulating leaf senescence. Large numbers of transcription factors in five stage-specific modules were identified by searching the Plant TF database (http://planttfdb.cbi.pku.edu.cn/, accessed on 17 May 2022). In the MEbrown module, a total of 44 transcription factors of 23 family types were identified, such as BGIOSGA002618 (bHLH family), BGIOSGA026208 (MYB family), and BGIOSGA022006 (NAC family) (Supplementary Table S5). In the MEmagenta module, four transcription factors were identified, including BGIOSGA013533 (bHLH family), BGIOSGA014498 (B3 family), BGIOSGA025611 (MYB_related family), and BGIOSGA020427 (ZF-HD family) (Supplementary Table S5). In the MEturquoise module, 78 transcription factors of 20 family types were identified, such as BGIOSGA013533 (bHLH family), BGIOSGA020819 (MYB family), and BGIOSGA020778 (WRKY family) (Supplementary Table S5). In the MEpink module, 13 transcription factors of nine family types were identified, such as BGIOSGA019054 (MYB family) and BGIOSGA026934 (NAC family) (Supplementary Table S5). In the MEblack module, 84 transcription factors of 21 family types were identified, such as BGIOSGA000374 (NAC family), BGIOSGA000430 (bZIP family), and BGIOSGA003013 (bHLH family) (Supplementary Table S5).

### 2.3. Identification of Senescence-Related Genes in Five Stage-Specific Modules

**Figure 3:**
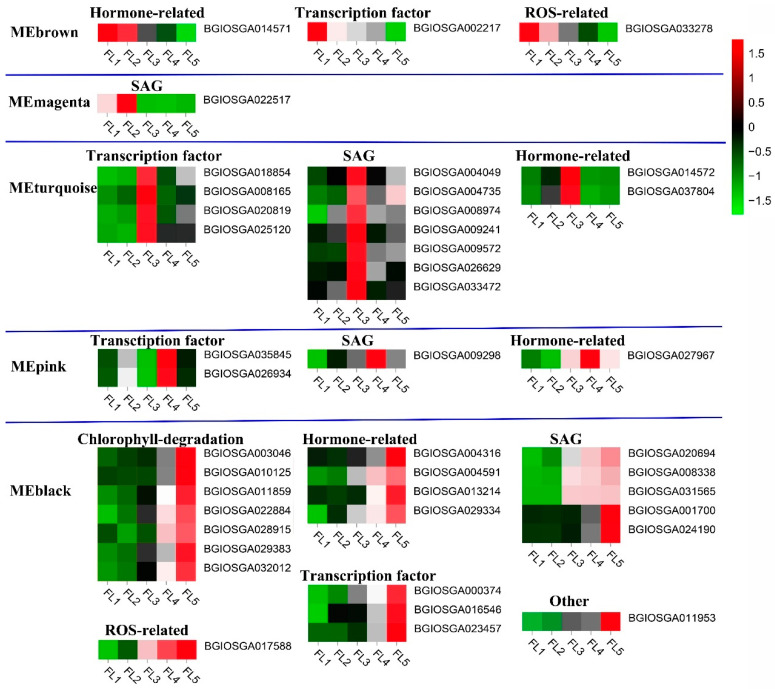
Heatmap analysis of senescence-related genes identified in each module (MEbrown, MEmagenta, MEturquoise, MEpink, MEblack) at different developmental stages (FL1–FL5). The expression value was log2 transformed, and color value ranged from −1.5 (green color, low expression) to 1.5 (red color, high expression). Furthermore, the gene name was displayed on the right of the heatmap, and the relevant biological processes in which the genes are involved are marked right above the heatmap, such as hormone-related, ROS-related, and chlorophyll-degradation-related.

Considering the key roles of senescence-related genes in leaf senescence, an analysis was performed to determine the existence of functionally validated senescence-related genes in this study. The results showed that three senescence-related genes were identified in the MEbrown module, including hormone-related gene BGIOSGA014571, transcription factor BGIOSGA002217, and BGIOSGA033278 (Figure 3; Table 1). In the MEmagenta module, senescence-associated gene (SAG) BGIOSGA022517 was identified (Figure 3; Table 1). In the MEturquoise module, seven SAGs (BGIOSGA004049, BGIOSGA004735, BGIOSGA008974, BGIOSGA009241, BGIOSGA009572, BGIOSGA026629, and BGIOSGA033472) were identified, four transcription factors (BGIOSGA008165, BGIOSGA018854, BGIOSGA020819, and BGIOSGA025120), and two hormone-related genes (BGIOSGA014572 and BGIOSGA037804) were identified (Figure 3; Table 1). In the MEpink module, two transcription factors (BGIOSGA035845 and BGIOSGA026934), a hormone-related BGIOSGA027967, and an SAG BGIOSGA009298 were identified (Figure 3; Table 1). In the MEblack module, five SAGs (BGIOSGA020694, BGIOSGA008338, BGIOSGA031565, BGIOSGA001700, BGIOSGA024190), three transcription factors (BGIOSGA000374, BGIOSGA016546, BGIOSGA023457), seven chlorophyll-degradation genes (BGIOSGA003046, BGIOSGA010125, BGIOSGA011859, BGIOSGA022884, BGIOSGA028915, BGIOSGA029383, and BGIOSGA032012), four hormone-related genes (BGIOSGA004316, BGIOSGA004591, BGIOSGA013214, BGIOSGA029334), and ROS-related gene BGIOSGA017588 and BGIOSGA011953 were identified (Figure 3; Table 1). All expression profiles of senescence-related genes were visualized by a heatmap (Figure 3).

### 2.4. Identification and Visualization of Hub Genes

**Figure 4:**
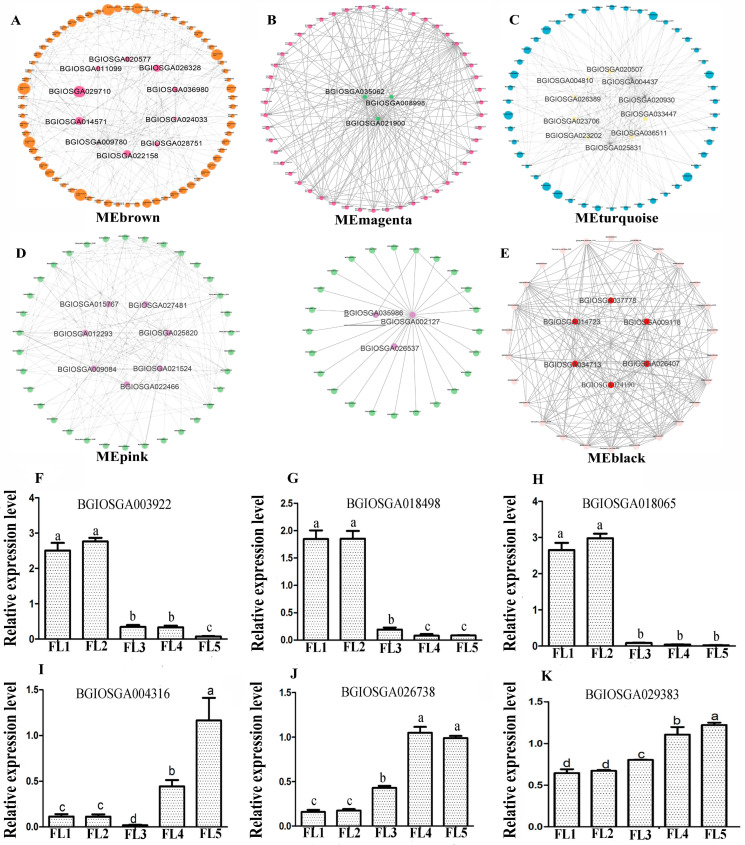
Hub gene interaction networks in five stage-specific modules and qPCR validation. The hub genes are placed in the center of the circle with an enlarged font and different colors. (**A**) Hub genes (pink color) in the center of the MEbrown module. (**B**) Hub genes (green color) in the center of the MEmagenta module. (**C**) Hub genes (yellow color) in the center of the MEturquoise module. (**D**) Hub genes (purple color) in the center of the MEpink module. (**E**) Hub genes (red color) in the center of the MEblack module. (**F**–**K**) Expression levels of six randomly selected DEGs in modules according to qRT-PCR. The values on the Y-axis show the relative expression levels. The X-axis shows five samples (FL1–FL5). Data are presented as mean ± SD (n = 3). Different lowercase letters (a, b, c, d) indicate a significant difference (*p* < 0.05), determined using one-way ANOVA with Tukey’s *t* test.

Hub genes were considered to possess the highest connectivity in a module, which has significant biological significance. In this study, the first 200 connections of the top 150 genes in each stage-specific module were analyzed to identify key hub genes, and visualized by using Cytoscape software (v3.10.3). The results showed that high-connectivity degree genes involved in the carbohydrate metabolic process (BGIOSGA009780, BGIOSGA024033 and BGIOSGA026328), lipid metabolic process (BGIOSGA011099), cytokinin (BGIOSGA014571) and five transcription factors (BGIOSGA028751, BGIOSGA029710, BGIOSGA036980, BGIOSGA020577, BGIOSGA022158) were considered as hub genes in the MEbrown module (Figure 4A; Supplementary Table S6).

In the MEmagenta module, high-connectivity degree hub genes (BGIOSGA021900, BGIOSGA035062, BGIOSGA008998) were functionally unknown (Figure 4B; Supplementary Table S6).

In the MEturquoise module, high-connectivity degree genes involved in protein phosphorylation (BGIOSGA004437, BGIOSGA020930, BGIOSGA036511), ABC transporter BGIOSGA025831, lipid metabolic process BGIOSGA023202, carbohydrate metabolic process BGIOSGA004810, oxidation reduction process BGIOSGA026389 and three transcription factors (BGIOSGA033447, BGIOSGA020507, BGIOSGA023706) were hub genes in the MEturquoise module (Figure 4C; Supplementary Table S6).

In the MEpink module, high-connectivity degree genes involved in the oxidation reduction process BGIOSGA025820, lipid metabolomic process BGIOSGA002127, heat shock proteins (BGIOSGA012293, BGIOSGA009084, BGIOSGA022466, BGIOSGA021524, BGIOSGA015767), and transcription factors (BGIOSGA026537, BGIOSGA035986, BGIOSGA027481) were identified to be hub genes (Figure 4D; Supplementary Table S6).

In the MEblack module, high-connectivity degree genes BGIOSGA014723, BGIOSGA024190 and four transcription factors (BGIOSGA026407, BGIOSGA034713, BGIOSGA037778, BGIOSGA009118) were identified to be hub genes (Figure 4E; Supplementary Table S6).

To furthermore validate the expression levels of DEGs, qRT-PCR was performed. The results showed that down-expressed DEGs (BGIOSGA003922, BGIOSGA018498, BGIOSGA018065) and up-expressed DEGs (BGIOSGA004316, BGIOSGA026738, BGIOSGA029383) were consistent with the RNA-Seq data (Figure 4F–K).

### 2.5. Integrative Transcriptome and Metabolome Analysis

**Figure 5:**
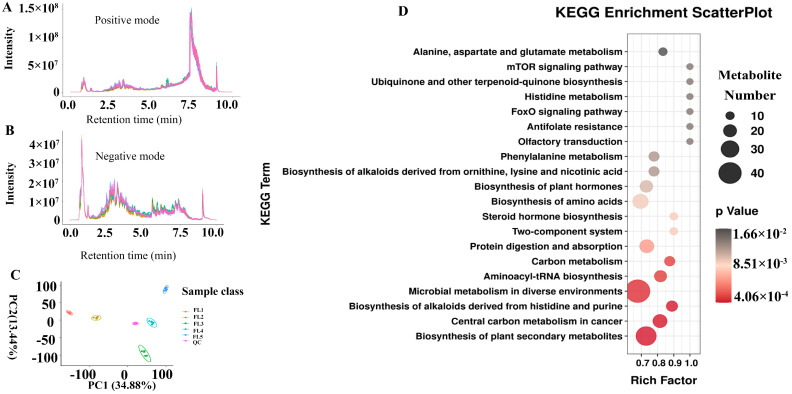
Metabolomic profile analysis. Detection of metabolites in positive ion modes (**A)** and negative ion modes (**B**). The horizontal axis represents the retention time, while the vertical axis represents intensity. (**C**) Principal component analysis (PCA) results of all samples (FL1–FL5), QC represents the quality control sample. Percentages are the variance explained by each principal component. (**D**) KEGG enrichment analysis of differential metabolites (DMs). The horizontal axis represents the rich factor, while the vertical axis represents the enriched pathway name. The color scale indicates different thresholds of the *p* value, and the size of the dot indicates the number of metabolites corresponding to each pathway. The top 20 pathways ranked by *p*-value are displayed.

To further understand which metabolites were associated with rice leaf senescence, untargeted metabolome analysis (LC-MS/MS) was performed in five developmental stages. In this study, the total ion chromatogram was analyzed in the positive ion mode and negative ion modes (Figure 5A,B). The result of principal component analysis (PCA) suggested that the metabolites were significantly separated and there was a large variation in leaf metabolites among the five stages (Figure 5C). According to the screening criteria (ratio ≥ 2 or ≤0.5, q value < 0.05, and VIP ≥ 1), a total of 380 differential metabolites (DMs) were identified (Supplementary Table S7). KEGG analysis showed that DMs were significantly enriched in metabolomic pathways, such as “Alanine, aspartate and glutamate metabolism”, “Phenylalanine metabolism”, “Biosynthesis of plant hormones”, “Protein digestion and absorption”, “Carbon metabolism”, and “Biosynthesis of alkaloids derived from histidine and purine” (Figure 5D).

**Figure 6:**
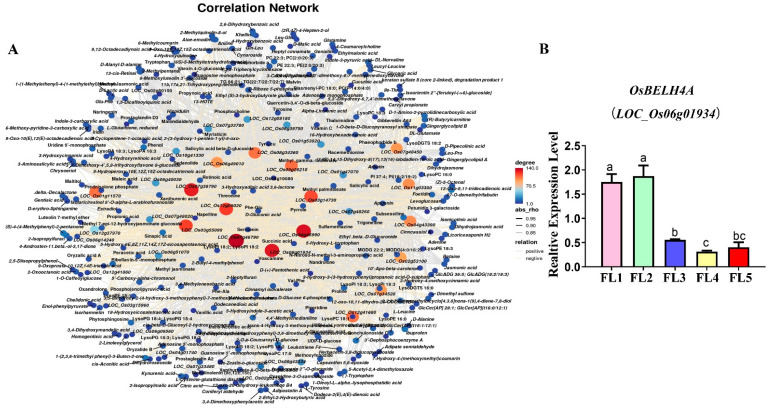
(**A**) The correlation network between 40 differentially expressed genes (DEGs) and 309 differential metabolites (DMs). Different shades of color signify connectivity degree. Specifically, the darker the color, the larger the number of metabolites linked to the gene. In this study, the absolute value of the correlation coefficient exceeds 0.8. A “positive” label indicates a positive correlation connected by solid lines, while negative correlations are connected by dashed lines. The top seven genes with high connectivity are marked in dark orange. (**B**) The expression level of *OsBELH4A*. The values on the Y-axis show the relative expression levels. The X-axis shows five samples (FL1–FL5). Data are presented as mean ± SD (n = 3). Different lowercase letters (a, b, c) indicate a significant difference (*p* < 0.05), determined using one-way ANOVA with Tukey’s *t* test.

Similarly to the WGCNA of the DEGs, the DMs were clustered into 13 modules using the WGCNA R package (v1.72) (Supplementary Figure S3). Based on the correlation coefficient between modules and traits, five modules (brown, red, green, blue, and turquoise) were considered highly related to leaf senescence, which comprise 309 DMs, including 103 lipids and lipid-like molecules; 36 phenylpropanoids and polyketides; 3 lignans, neolignans and related compounds; 25 organic acids and derivatives; 48 organic acids and derivatives; 23 benzenoids; 4 alkaloids and derivatives; 6 nucleosides, nucleotides, and analogs; 31 organoheterocyclic compounds and 30 unknown metabolites (Supplementary Table S8). Furthermore, correlation analysis was conducted between 40 hub genes and 309 DMs, and the results showed that the 40 hub genes were strongly correlated with 262 DMs, resulting in a total of 2280 pairs of correlation relationships (Supplementary Table S9). Finally, the correlation network was visualized (Figure 6A). Considering the high-connectivity degree (Supplementary Table S6) and strong correlation coefficient |rho| ≥ 0.8, the top seven genes with high connectivity are marked in dark orange. Additionally, the expression trend of BGIOSGA022158 (*OsBELH4A*, *LOC_Os06g01934*) gene was down-regulated, which was consistent with the RNA-Seq results, thus considered a candidate gene for functional verification (Figure 6B).

### 2.6. Overexpression of OsBELH4A Gene Delayed Leaf Senescence

**Figure 7:**
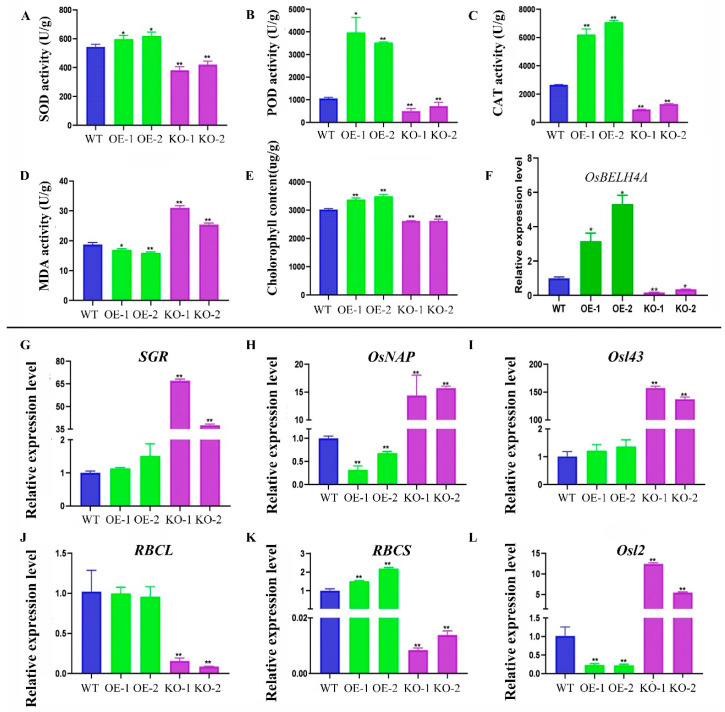
Detection of physiological indicators and senescence marker genes in transgenic rice. Statistical analysis of SOD activity (**A**), POD activity (**B**), CAT activity (**C**), MDA content (**D**), chlorophyll content (**E**), and *OsBELH4A* expression level (**F**) between wild type (WT) and transgenic lines (OE and KO). (**G**–**L**) Expression levels of senescence marker genes (*SGR*, *OsNAP*, *Osl43*, *RBCL*, *RBCS*, *Osl2*). The data are expressed as the mean ± SD (n = 3). Statistical significance was defined using Student’s *t*-test: *p* < 0.05 (*) and *p* < 0.01 (**).

In this study, the transgenic overexpression rice lines (OE-1, OE-2) and knockout lines (KO-1, KO-2) were obtained after T2 generation. The qPCR result showed that the relative expression level of the *OsBELH4A* gene in the OE lines was significantly higher than that in the WT lines, while that in the KO lines was lower (Figure 7F). Leaf senescence usually accompanies the accumulation of reactive oxygen species. The result showed that scavenging enzyme activity (SOD, POD and CAT) in the OE lines was significantly higher than that in the WT while it displayed opposite trends in the KO lines. In addition, the content of MDA in the OE lines was lower than that in the WT, while that in the KO lines was higher (Figure 7A–D). Most importantly, the degradation of chlorophyll implied the initiation of leaf senescence. In this study, the content of chlorophyll in the OE lines significantly increased than that in the WT lines, while it significantly decreased in the KO lines (Figure 7E).

**Figure 8:**
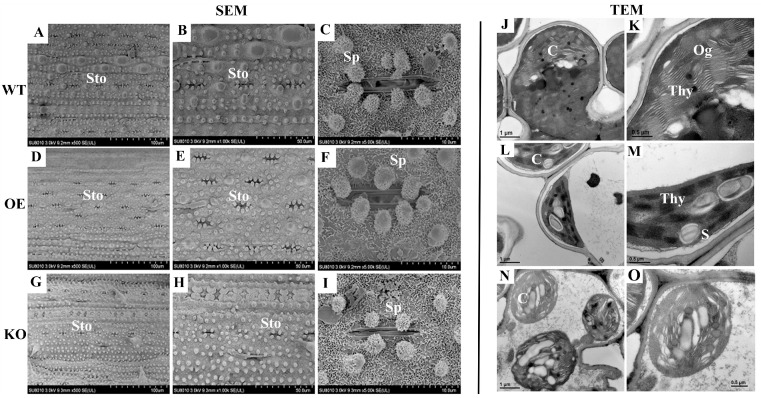
Electron microscope observation of flag leaf in rice. (**A**–**C**,**J**,**K**) WT lines, (**D**–**F**,**L**,**M**) overexpression lines (OE), (**G**–**I**,**N**,**O**) CRISP/Cas9 lines (KO). Left: SEM; right: TEM. (**A**,**D**,**G**), bar = 100 µm; (**B**,**E**,**H**), bar = 50 µm; (**C**,**F**,**I**), bar = 10 µm; Sto: stomata; Sp: silicified protrusion. (**J**,**K**,**N**), bar = 1 µm; (**K**,**M**,**O**), bar = 0.5 µm; C: chloroplast; Thy: thylakoid; S: starch granules; Og: osmophilic granules.

The expression level of senescence marker genes can also reflect the degree of leaf senescence. In the KO lines, the chlorophyll-degradation-related genes (*SGR*, *OsNAP*) and two SAGs (*Osl43*, *Osl2*) were significantly up-regulated, while photosynthetic system-related genes (*RBCL*, *RBCS*) were significantly down-regulated. By contrast, the opposite trend was displayed in the OE lines (Figure 7G–L). Furthermore, the SEM result showed that the stomatal aperture was smaller and the number of silicified protrusions around the stomata were significantly reduced in the KO lines, while that in the OE lines were the opposite (Figure 8). The TEM result showed that the thylakoid structure in the KO lines was more loosely arranged, and the chloroplast was underdeveloped (Figure 8). These results suggested that overexpression of the *OsBELH4A* gene delayed leaf senescence.

### 2.7. OsBELH4A Gene Affected Rice Yield Traits in Varying Degrees

**Figure 9:**
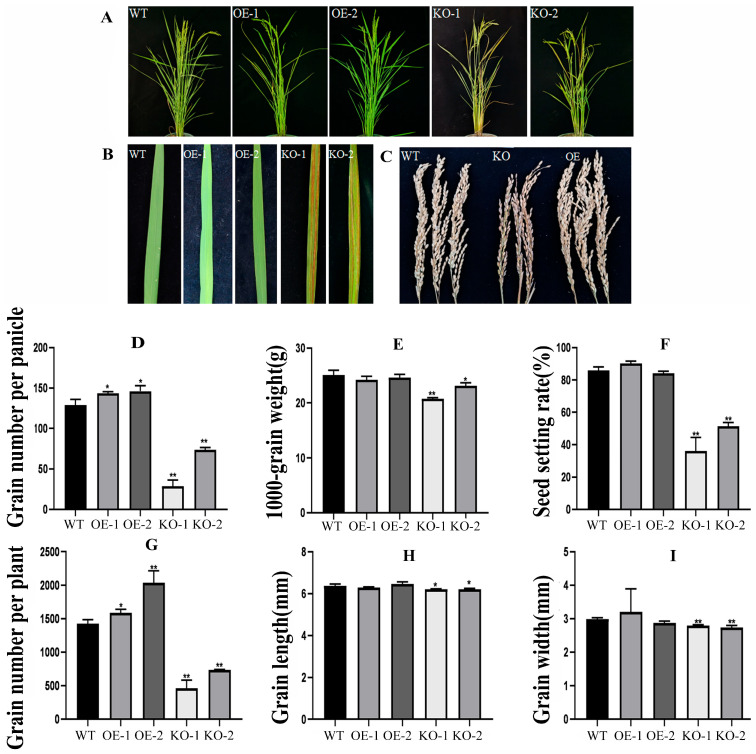
Investigation of leaf phenotype and agronomic traits. (**A**) The phenotype of whole rice lines WT and transgenic rice (OE and KO) in the flowering stage. (**B**) The flag leaf phenotype of WT and transgenic rice (OE and KO) in the flowering stage. (**C**) The harvested panicle. (**D**–**I**) Statistic analysis of agronomic traits including grain number per panicle, 1000-grain weight, seed setting rate, grain number per plant, grain length and grain width among the WT, OE, and KO lines. The data are expressed as the mean ± SD (n = 30). Statistical significance was defined using Student’s *t*-test: *p* < 0.05 (*) and *p* < 0.01 (**).

To explore the effect of the *OsBELH4A* gene on the yield traits, the yield-related parameters such as grain number per plant, grain number per panicle and 1000-grain weight were investigated. The rice was planted in the experimental field, and the leaf phenotype was observed in the entire growth period. The result showed that the KO lines showed a yellowing phenotype with leaf spots, shorter plant height and fewer tiller numbers (Figure 9A,B). The plants were harvested until the maturity stage (Figure 9C) and were statistically analyzed. The result suggested that the number of grains per plant, spike length, grains per panicle and plant height in the OE lines significantly increased to varying degrees, while no significant changes were observed with 1000-grain weight, seed setting rate, effective tillering, grain length, and grain width (Figure 9D–I). By contrast, all the indicators of yield traits were significantly reduced in the KO lines. (Figure 9D–I). These results implied the positive potential of the *OsBELH4A* gene in rice yield.

### 2.8. OsBELH4A Gene Expression Responded to Hormone SA and Auxin

**Figure 10:**
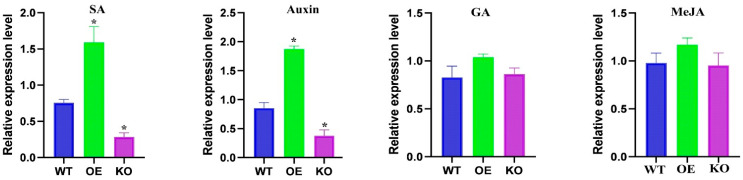
Expression levels of the *OsBELH4A* gene with application of different exogenous phytohormones (SA, GA, MeJA, Auxin) in wild-type and transgenic rice lines. The data are expressed as the mean ± SD (n = 3). Statistical significance was defined using Student’s *t*-test: *p* < 0.05 (*).

To demonstrate which hormonal pathway *OsBELH4A* is involved in regulating leaf senescence and yield traits, four phytohormones (GA, SA, MeJA, 2,4-D) were applied at the seedlings stage after the cis-acting element analysis by using the PlantCARE database. After the application of GA and MeJA, there was no significant difference in the expression level of *OsBELH4A* in the transgenic lines compared with that in the wild type (Figure 10). Surprisingly, the application of hormones SA and auxin significantly elevated the expression level of *OsBELH4A* in the OE lines while they displayed opposite trends in the KO lines (Figure 10). These results implied that *OsBELH4A* played essential roles in regulating leaf senescence and might participate in SA and auxin metabolic pathway.

## 3. Discussion

### 3.1. The Roles of Genes Related to Carbohydrate and Lipid Metabolism in the Leaf Senescence of Rice

Studies have shown that carbohydrates and lipids not only provided energy substances required for plant growth and development, but also played roles in the process of leaf senescence [62]. The *Arabidopsis thaliana sweetie* mutant had defects in the carbohydrate metabolic pathway, which in turn affected plant growth, development, and senescence process [63]. During leaf senescence, carbohydrate change in the leaf acted as a regulatory factor for chloroplast autophagic degradation through ribulose-1,5-bisphosphate carboxylase [64]. Lipid turnover controlled the energy density and nutrient content of crops [65]. During leaf senescence, significant changes occurred in carbohydrate and lipid metabolism [66]. In this study, the lipid metabolic process (GO:0006629) and carbohydrate metabolic process (GO:0005975) were significantly enriched (Figure 2). In addition, three (BGIOSGA009780, BGIOSGA024033, BGIOSGA026328) and two hub genes (BGIOSGA004810 and BGIOSGA014723) were annotated to be involved in carbohydrate metabolism (Figure 4A,C). Hub genes BGIOSGA011099, BGIOSGA023202 and BGIOSGA002127 were involved in lipid metabolism (Figure 4A,C,D). These results implied the roles of genes related to carbohydrate and lipid metabolism in the leaf senescence of rice.

### 3.2. The Roles of Transcription Factors in the Leaf Senescence of Rice

Transcription factors, such as NAC-type, WRKY-type, and MYB-type, have been reported to play roles in the process of leaf senescence [67]. In this study, five stage-specific modules were obtained through WGCNA, and 10 transcription factors related to leaf senescence were identified (Table 1). For example, the *OsWRKY42* overexpression line showed a premature senescence, accompanied by the accumulation of ROS and a decrease in chlorophyll content [37]. In this study, the gene BGIOSGA008165 (*LOC_Os02g26430*) was identified as *OsWRKY42* (Table 1). The T-DNA insertion mutant *OsWRKY5* can promote leaf senescence; conversely, *OsWRKY5*-RNAi delayed leaf senescence [41]. In this study, the gene BGIOSGA018854 (*LOC_Os05g04640*) was identified as OsWRKY5 transcription factor (Table 1). In addition, an MYB-type transcription factor *OsMYB102* can delay leaf senescence [42]. In this study, the gene BGIOSGA020819 (*LOC_Os06g43090*) was identified as *OsMYB102* (Table 1). The gene *OsHox33* has been identified as a transcription factor. Reducing the expression of *OsHox33* through RNAi can accelerate leaf senescence in rice [43]. In this study, the gene BGIOSGA035845 (*LOC_Os12g41860*) was identified as *OsHox33* (Table 1). The novel nuclear CCCH-zinc finger protein transcription factor *OsDOS* showed delayed leaf senescence, while the RNAi line caused accelerated leaf senescence [36]. In this study, the gene BGIOSGA002217 (*LOC_Os01g09620*) was identified as *OsDOS* (Table 1).

Furthermore, rice *OsNAC2* [38], *ONAC011* [39], *ONAC096* [40], *ONAC106* [45], and *OsNAC6* [44] NAC transcription factors were found to regulate leaf senescence. In this study, BGIOSGA016546 (*LOC_Os04g38720*), BGIOSGA023457 (*LOC_Os06g46270*), BGIOSGA025120 (*LOC_Os07g04560*) and BGIOSGA000374 (*LOC_Os01g66120*) genes were identified as *OsNAC2*, *ONAC011*, *ONAC096*, *ONAC106*, and *OsNAC6*, respectively (Table 1). In addition, the biological process “regulation of transcription, DNA-templated (GO:0006355)” was significantly enriched (Figure 2). In this study, 16 hub genes were identified to be transcription factors (Figure 4). These results implied the roles of genes in the leaf senescence of rice through transcriptional regulation.

### 3.3. The Roles of Hormones in the Leaf Senescence of Rice

Hormones, such as cytokinin, jasmonic acid (JA), and abscisic acid (ABA), were reported to play roles in leaf senescence [68]. In this study, eight hormone-related genes were identified (Table 1). Some studies have identified that cis-zeatin O-glucosyltransferases (*cZOGT1*, *cZOGT2*) genes resulted in short branches, reduced crown roots, and delayed leaf senescence [51]. In this study, the genes BGIOSGA014572 (*LOC_Os04g46980*) and BGIOSGA014571 (*LOC_Os04g46990*) were identified as *cZOGT1* and *cZOGT2*, respectively (Table 1). Meanwhile, *cZOGT2* was also identified as a hub gene (Figure 4A). The ethylene signaling pathway gene *OsRTH1* overexpression line can prevent ethylene-induced changes in growth and development including leaf senescence [50]. The identified gene BGIOSGA004316 (*LOC_Os01g51430*) in this study was identified as *OsRTH1* (Table 1). JA and its derivatives have been reported to play roles in the process of leaf senescence. For example, overexpression of the *OsPME1* gene can accelerate leaf senescence and chlorophyll degradation, while the *OsPME1*-RNAi line showed retarded leaf senescence and chlorophyll degradation [48]. The identified gene BGIOSGA004591 (*LOC_Os01g57854*) in this study was identified as *OsPME1* (Table 1).

Moreover, the tryptophan decarboxylase-encoding gene *TDC* is a key gene in the serotonin biosynthesis pathway. Transgenic lines overexpressing *TDC* delayed leaf senescence, while *TDC*-RNAi lines accelerated leaf senescence [53]. In this study, the gene BGIOSGA027967 (*LOC_Os08g04540*) was identified as *TDC1* (Table 1). Auxin is also a type of plant hormone that plays a crucial role in regulating plant senescence [69]. Overexpression of the auxin gene *SAUR39* led to a lower chlorophyll content, faster leaf senescence, and lower yield in transgenic rice lines [49]. The identified gene BGIOSGA029334 (*LOC_Os09g37330*) in this study was identified as *SAUR39* (Table 1).

It has been reported that overexpression of the *OsNCED3* and *OsNCED5* genes can promote leaf senescence and increase the content of abscisic acid (ABA) [46,47]. In this study, the genes BGIOSGA013214 (*LOC_Os03g44380*) and BGIOSGA037804 (*LOC_Os12g42280*) were identified as *OsNCED3* and *OsNCED5*, respectively (Table 1). Furthermore, large numbers of genes were significantly enriched in the biological process “response to hormone (GO:0009725)” (Figure 2). In addition, the phytohormone treatment also elevated the expression levels of the *OsBELH4A* gene (Figure 10), which may imply the roles of hormones in the leaf senescence of rice.

### 3.4. The Roles of Chlorophyll Degradation-Related Genes in the Leaf Senescence of Rice

The most obvious sign of the onset of leaf senescence in rice is leaf yellowing, which is accompanied by chlorophyll degradation. Currently, large numbers of genes related to chlorophyll degradation have been identified. For example, both the mutation of *nyc1* (non-yellow coloring1) and *nyc3* in rice resulted in a stay-green phenotype [13,53]. The *nol* mutant showed a stay-green trait. It can interact with NYC1 and form a complex to perform the function of chlorophyll b reductase [14]. The *sgr* mutant showed a stay-green phenotype, and overexpression of the *SGR* gene can accelerate chlorophyll degradation [16]. In this study, the genes BGIOSGA003046 (*LOC_Os01g12710*), BGIOSGA022884 (*LOC_Os06g24730*), BGIOSGA010125 (*LOC_Os03g45194*), and BGIOSGA029383 (*LOC_Os09g36200*) were identified as *NYC1*, *NYC3*, *NOL*, and *SGR*, respectively (Table 1).

In addition, it has been reported that the leaves of *PAO*-RNAi transgenic plants died before regeneration; silencing of the *RCCR1* gene led to lesion-mimic leaf spots and premature leaf death [54]. In this study, the genes BGIOSGA011859 (*LOC_Os03g05310*) and BGIOSGA032012 (*LOC_Os10g25030*) were identified as *PAO* and *RCCR1*, respectively (Table 1).

Moreover, the nonsense mutation of gene *OsAkaGal* resulted in delayed leaf senescence. Transgenic lines overexpressing *OsAkaGal* showed retarded growth and a light-green phenotype [55]. In this study, the gene BGIOSGA028915 (*LOC_Os08g38710*) was identified as *OsAkaGal* (Table 1). These results implied that the chlorophyll degradation pathway played roles in the leaf senescence of rice.

### 3.5. The Roles of ROS-Related Genes in the Leaf Senescence of Rice

As is well known, reactive oxygen species (ROS) are closely related to leaf senescence [70]. A reactive oxygen species-sensitive leaf senescence 1 (*rls1*) mutant showed premature leaf senescence, accumulated H_2_O_2_ and increased superoxide dismutase (SOD) activity [59]. In this study, the gene BGIOSGA017588 (*LOC_Os05g48390*) was identified as *RLS1* (Table 1). In addition, GO enrichment in this study showed that large numbers of genes were significantly enriched in the “oxidation–reduction process (GO:0055114)” (Figure 2). Most importantly, hub genes BGIOSGA026389 and BGIOSGA025820 were annotated to be involved in the oxidation reduction process (Figure 4C,D). It has been reported that heat shock protein was involved in the ROS metabolism process [71]. In this study, five hub genes BGIOSGA012293 (*LOC_Os03g15960*), BGIOSGA009084 (*LOC_Os02g52150*), BGIOSGA022466 (*LOC_Os06g09560*), BGIOSGA021524 (*LOC_Os06g14240*) and BGIOSGA015767 (*LOC_Os04g01740*) encoded 17.9 KDa, 24.1 KDa, DnaJ, 16.0 KDa and Hsp82 heat shock proteins, respectively (Figure 4). These results implied the roles of ROS metabolism in the leaf senescence of rice.

### 3.6. OsBELH4A Negatively Affected Leaf Senescence by Regulating Chlorophyll and ROS

Chlorophyll played central roles in photosynthesis. *OsBELH4A* gene knockout lines displayed the leaf-spot phenotype (Figure 9A,B). The chlorophyll level in *OsBELH4A* KO lines was significantly down-regulated, while that in the OE lines was up-regulated to varying degrees (Figure 7E). Furthermore, the results of SEM and TEM indicated the occurrence of leaf senescence (Figure 8).

The massive production of ROS is one of the earliest responses of plants to stresses such as senescence and can directly kill cells [72]. In this study, SOD, CAT and POD in the KO lines were significantly reduced, and the content of MDA was significantly increased. Unsurprisingly, the trends were opposite in the OE lines (Figure 7A–D). Investigation results of agronomic traits showed that the total number of grains per plant, panicle length, number of grains per panicle, and plant height in *OsBELH4A*-OE lines increased to varying degrees, while those significantly decreased in the KO lines, which implied the negative roles of the *OsBELH4A* gene (Figure 9). These results suggested that *OsBELH4A* regulated leaf senescence by affecting chlorophyll and ROS, thereby regulating rice yield traits.

### 3.7. OsBELH4A Negatively Affected Leaf Senescence by Regulating the Expression Levels of SAGs and Chlorophyll Degradation-Related and Photosynthetic System-Related Genes

*OsNAP* is an important component of the plant senescence signaling pathway. It is specifically induced by abscisic acid, the targeted regulation of chlorophyll degradation genes (*SGR*, *NYC1*, *NYC3*, and *RCCR1*) and nitrogen transport genes to accelerate leaf senescence [9]. *SGR* encoded chloroplast stay-green protein 1, which affected the degradation of chlorophyll and pigment protein complexes by regulating the activity of pheophorbide a oxygenase. Therefore, its mutant showed chlorophyll retention [73]. Two senescence marker genes *Osl43* and *Osl2* were significantly up-regulated in premature leaf senescence mutants [74]. *RBCL* and *RBCS* are two typical leaf senescence down-regulated genes, encoding the large and small subunits of ribulose-1,5-bisphosphate carboxylase/oxygenase in chloroplasts, respectively. They are key genes in the carbon reaction of photosynthesis [75,76,77]. In this study, the chlorophyll degradation-related genes (*SGR*, *OsNAP*) and SAG (*Osl43*, *Osl2*) are significantly up-regulated, while photosynthetic system-related genes (*RBCL*, *RBCS*) are significantly down-regulated in the KO lines (Figure 7G-L). In the OE lines, the opposite trends were observed (Figure 7G-L), which implied that the *OsBELH4A* gene negatively affected leaf senescence by regulating the expression levels of SAGs and chlorophyll degradation-related and photosynthetic system-related genes.

## 4. Materials and Methods

### 4.1. Plant Material and Phenotypic Characterization of Senescence Stages

Rice cultivar 93-11 (*Oryza sativa* L. subsp. *indica*) was cultivated in a paddy field. According to the phenotypic characteristic of leaves, the rice flag leaves at booting stage (FL1, 9 days before flowering), flowering stage (FL2, 3 days after flowering), early-senescence stage (FL3, 9 days after flowering), mid-senescence stage (FL4, 19 days after flowering), and late-senescence stage (FL5, 29 days after flowering) were collected, respectively (Supplementary Figure S1A,B). To effectively examine the characterization of leaf senescence, the chlorophyll content per gram of leaf fresh weight (FW) was determined (Supplementary Figure S1C). Furthermore, the expression levels of senescence-marker genes *OsNAP* (*LOC_Os03g21060*) and *SGR* (*LOC_Os09g36200*) were performed by quantitative real-time PCR (qPCR) (Supplementary Figure S1D,E).

### 4.2. Transcriptome Data Collection, Differential Expression and Weighted Gene Co-Expression Network Analysis (WGCNA)

After confirming the leaf senescence stages, the leaves were used for transcriptome sequencing and the wide-transcriptome raw data including mRNAs were collected at the Genome Sequence Archive database under accession number CRA003505 (URL: http://bigd.big.ac.cn/gsa/s/YB3P26qq, accessed on 17 May 2022) [78]. The clean reads derived from transcriptome raw reads were mapped to the rice reference genome using HISAT2 software (v2.0.5) [79], followed by assembly with StringTie software (v2.2.3) [80]. Next, the assembled transcripts were annotated using the gffcompare program. Finally, the protein-coding genes were identified. The differentially expressed genes (DEGs) were identified with an absolute value of log2(fold change) > 1 and a false discovery rate (FDR) < 0.05.

To further identify DEGs related to leaf senescence, a weighted gene co-expression network analysis (WGCNA) of DEGs was conducted using the WGCNA R package (v1.72) [81]. An unsigned co-expression relationship was built based on the adjacency matrix. The one-step network construction and module detection were adopted using the “dynamic hybrid tree cut algorithm” with a power value of 5, minimum module size of 30, and merge cut height of 0.2826. The other parameters were defined as default values. Highly similar modules were subsequently identified by clustering and then merged into new modules on the basis of eigengenes. The correlation of each module was also analyzed and visualized by a heatmap. Finally, the co-expression network was visualized by Cytoscape software (v3.10.3).

### 4.3. Quantitative Real-Time PCR Validation of Selected DEGs

For the expression validation, six DEGs were randomly selected from RNA-Seq data to analyze the relative gene expression levels. After cDNA synthesis, the quantitative real-time PCR (qPCR) was performed using RNA-specific primer with the SYBR Green PCR Kit. Three genes *UBC* (*LOC_Os02g42314*), *ARF* (*LOC_Os05g41060*), and *Profilin-2* (*LOC_Os06g*05880) were used as internal reference genes to normalize the qPCR data for mRNAs. The 2^−ΔΔCT^ method was used to calculate relative expression levels of the genes [82]. The reaction was carried out using three biological replicates with three technical replicates. All pairs of primers used for qPCR are listed in Supplementary Table S10.

### 4.4. Metabolome Analysis

The collected samples (FL1–FL5) at five stages were used for metabolome analysis and six biological replicates were performed. Flag leaves were ground into powder using liquid nitrogen and dissolved into 50% methanol solution. After precipitation overnight at −20 °C, the extracted metabolites were analyzed by an ultra-high-performance liquid chromatography tandem mass spectrometry system equipped with an Acquity UPLC BEH C18 column (1.8 μm, 2.1 × 100 mm) (Waters, Milford, MA, USA). The analysis was performed in positive–negative ionization mode to ensure comprehensive detection of metabolic data. Principal component analysis (PCA), hierarchical cluster analysis (HCA), and Pearson’s correlation coefficients (PCCs) were used to evaluate metabolite data. Differential metabolites (DMs) were determined by applying specific thresholds: variable importance in projection value (VIP) ≥ 1, fold change > 2 or <0.5, and a significance level of *p* ≤ 0.05. The identified metabolites were annotated using the KEGG database (http://www.kegg.jp/kegg/compound/, accessed on 17 May 2022), and annotated metabolites were then mapped to the KEGG Pathway database (http://www.kegg.jp/kegg/pathway.html, accessed on 17 May 2022) for further analysis.

### 4.5. Gene Cloning, Vector Construction and Rice Transformation

To generate an *OsBELH4A* overexpression construct, the CDS of *OsBELH4A* was amplified from the ZH11 genome with a specific primer (F: 5′-cagtCGTCTCacaacatgatggcggcccatcatca-3′, R: 5′-cagtCGTCTCatacattagctagctcctgcaaaat-3′). The CDS fragment was cloned into the pBWA (V) HS vector driven by the CaMV 35S promoter via the *Bsa*I/*BsmB*I restriction enzymes. To generate the *OsBELH4A*-knockout vector, the CRISPR/Cas9 targets within the *OsBELH4A* exons were designed using Cas-designer (target1: TCGTCGCCCCCAAACGCCAGCGG, target2: CAAGTACCTGGGCCCTGTGAAGG). The gRNA expression cassette was amplified with a specific primer (F: 5′-cagtggtctcatgcaTCGTCGCCCCCAAACGCCAG-3′, R: 5′-cagtggtctcaaa acTCACAGGGCCCAGGTACTTG-3′) and then inserted into the modified pYLCRISPRCas9Pubi-H-osu3 plasmid. After confirmation by sequencing, these resulting constructs were then transferred into ZH11 by the *Agrobacterium-mediated* method to generate transgenic rice plants as described previously [83]. Homozygous positive overexpression lines were identified after two generations. Homozygous Cas9 gene-editing lines were identified by PCR and sequencing. Real-time quantitative PCR (qPCR) was used to determine higher expression levels for selecting the overexpression lines and ensuring that the expression levels of the gene-editing lines were down-regulated. The cDNA used for qPCR was obtained in a uniform growth state from each line. The primers are shown in Supplementary Table S10.

### 4.6. Detection of Physiological Indicators and Senescence Marker Genes in Transgenic Rice

Chlorophyll content of the flag leaf was determined by extracting with acetone. The MDA content and ROS-scavenging related enzyme (SOD, CAT, and POD) activity in wild-type ZH11 and transgenic rice were measured using an Assay Kit (Beijing Solarbio Science & Technology Co., Ltd., Beijing, China). Furthermore, the expression levels of senescence marker genes *SGR* (*LOC_Os09g36200*), *OsNAP* (*LOC_Os03g21060*), *RBCL* (*LOC_Os10g21268*), *RBCS* (*LOC_Os12g19470*), *Osl43* (*LOC_Os01g24710*), and *Osl2* (*LOC_Os04g52450*) were quantified by qPCR (Supplementary Table S10).

### 4.7. Electron Microscopy Observation

For scanning electron microscope observation, the flag leaf samples were fixed overnight at 4 °C with 2.5% glutaraldehyde in 0.1 M phosphate buffer (pH7.4), and then the samples were dehydrated in a graded ethanol series. The dehydrated samples were critical-point-dried, sputter-coated with gold palladium in a Hitachi Model E-1010 ion sputter (Hitachi, Tokyo, Japan) for 4–5 min, and observed in a Hitachi Model SU-8010 (Hitachi, Japan) scanning electron microscope.

For transmission electron microscopy analysis, samples were fixed overnight at 4 °C with 2.5% glutaraldehyde in 0.1 M phosphate buffer (pH 7.4). After washing with phosphate buffer three times, samples were postfixed with 1% (*v*/*v*) OsO_4_ for 2 h. After dehydration and infiltration, the specimen was placed in an Eppendorf-contained Spurr resin and heated at 70 °C for more than 9 h. The specimen was sectioned in a LEICA EM UC7 ultratome and sections were stained by uranyl acetate and alkaline lead citrate for 5 to 10 min, and observed in a Hitachi Model H-7650 TEM. The procedure of SEM and TEM analysis was performed at the Bio-Ultrastructure Analysis Laboratory of Analysis Center of Agrobiology and Environmental Sciences (Zhejiang University, Hangzhou, China).

### 4.8. Investigation of Leaf Phenotype and Agronomic Traits

The uniformly growing plants of overexpressing lines, knockout lines, and wild-type plants were cultivated at a transgenic experimental field in Fuyang, Zhejiang Province, China. The distance between the plants within a row was 15 cm, and the distance between the rows was 20 cm. The leaf phenotype of the transgenic rice was observed throughout the growth period. Before harvest, several agronomic traits were measured including plant height, spike length, and effective tiller number per plant. Grain-related traits, including grain length, grain width, grain number per panicle, seed setting rate, 1000-grain weight, and grain number per plant, were measured after harvesting and stored at 37 °C for one week. Thirty individual plants in each line were used to evaluate significance.

### 4.9. Exogenous Phytohormone Treatment

After germination, the 4-week-old seedlings of transgenic plants and wild-type plants were subjected to different treatments. Phytohormone treatments were performed by cultivating in nutrient solutions with MeJA (0.1 mM), JA (0.1 mM), SA (0.1 mM), and 2,4-D (0.1 mM). Leaves were collected for total RNA isolation to analyze *OsBELH4A* expression after 12 h.

### 4.10. Statistical Analysis

Statistical analyses were performed using two-tailed Student’s *t*-test via GraphPad Prism version 8 (GraphPad Software Inc., San Diego, CA, USA). Quantitative data were expressed as means ± standard deviation (SD). Statistical significance was defined as *p* < 0.05 (*) and *p* < 0.01 (**); when comparing multiple groups, statistical significance was determined by one-way analysis of variance (ANOVA) with Tukey’s multiple comparisons test.

## Figures and Tables

**Table 1:** The identified senescence-related genes in five stage-specific modules by WGCNA.

Category	Gene ID	MSU-ID	Gene Name	Reference	Module
Transcription factors	BGIOSGA002217	*LOC_Os01g09620*	*OsDOS*	[36]	brown
	BGIOSGA008165	*LOC_Os02g26430*	*OsWRKY42*	[37]	turquoise
	BGIOSGA016546	*LOC_Os04g38720*	*OsNAC2*	[38]	black
	BGIOSGA023457	*LOC_Os06g46270*	*ONAC011*	[39]	black
	BGIOSGA025120	*LOC_Os07g04560*	*ONAC096*	[40]	turquoise
	BGIOSGA018854	*LOC_Os05g04640*	*OsWRKY5*	[41]	pink
	BGIOSGA020819	*LOC_Os06g43090*	*OsMYB102*	[42]	turquoise
	BGIOSGA035845	*LOC_Os12g41860*	*OsHox33*	[43]	turquoise
	BGIOSGA000374	*LOC_Os01g66120*	*OsNAC6*	[44]	pink
	BGIOSGA026934	*LOC_Os08g33670*	*ONAC106*	[45]	black
Hormone-related genes	BGIOSGA013214	*LOC_Os03g44380*	*OsNCED3*	[46]	black
	BGIOSGA037804	*LOC_Os12g42280*	*OsNCED5*	[47]	turquoise
	BGIOSGA004591	*LOC_Os01g57854*	*OsPME1*	[48]	black
	BGIOSGA029334	*LOC_Os09g37330*	*SAUR39*	[49]	black
	BGIOSGA004316	*LOC_Os01g51430*	*OsRTH1*	[50]	black
	BGIOSGA014572	*LOC_Os04g46980*	*cZOGT1*	[51]	turquoise
	BGIOSGA014571	*LOC_Os04g46990*	*cZOGT2*	[51]	brown
	BGIOSGA027967	*LOC_Os08g04540*	*TDC1*	[52]	pink
Chlorophyll degradation-related genes	BGIOSGA003046	*LOC_Os01g12710*	*NYC1*	[13]	black
	BGIOSGA010125	*LOC_Os03g45194*	*NOL*	[14]	black
	BGIOSGA022884	*LOC_Os06g24730*	*NYC3*	[53]	black
	BGIOSGA029383	*LOC_Os09g36200*	*SGR*	[16]	black
	BGIOSGA011859	*LOC_Os03g05310*	*PAO*	[54]	black
	BGIOSGA032012	*LOC_Os10g25030*	*RCCR1*	[54]	black
	BGIOSGA028915	*LOC_Os08g38710*	*OsAkaGal*	[55]	black
SAGs	BGIOSGA022517	*LOC_Os06g10560*		[56]	magenta
	BGIOSGA020694	*LOC_Os06g46160*		[57]	black
	BGIOSGA008338	*LOC_Os02g32520*		[57]	black
	BGIOSGA031565	*LOC_Os10g36848*		[57]	black
	BGIOSGA009298	*LOC_Os02g57280*		[57]	pink
	BGIOSGA004049	*LOC_Os01g44120*		[57]	turquoise
	BGIOSGA004735	*LOC_Os01g61460*		[56]	turquoise
	BGIOSGA009241	*LOC_Os02g56250*		[57]	turquoise
	BGIOSGA009572	*LOC_Os03g60090*		[57]	turquoise
	BGIOSGA026629	*LOC_Os08g41280*		[57]	turquoise
	BGIOSGA008974	*LOC_Os02g49650*		[56]	turquoise
	BGIOSGA033472	*LOC_Os10g41930*		[57]	turquoise
	BGIOSGA001700	*LOC_Os01g24710*		[57]	black
	BGIOSGA024190	*LOC_Os07g34520*		[58]	black
ROS	BGIOSGA017588	*LOC_Os05g48390*	*RLS1*	[59]	black
	BGIOSGA033278	*LOC_Os10g37180*	*OsGDCH*	[60]	brown
Other	BGIOSGA011953	*LOC_Os03g07530*	*OsFBK12*	[61]	black

## Data Availability

All data generated or analyzed during this study are included in this published article and its Supplementary Information files.

## Supplementary Materials

The following supporting information can be downloaded at https://www.mdpi.com/article/10.3390/plants14192973/s1, Figure S1. The senescence feature of rice flag leaves sampled at five developmental stages. (A) The whole rice plant at booting stage (FL1, before flag leaf senescence), flowering stage (FL2, before flag leaf senescence), early-senescence stage (FL3), mid-senescence stage (FL4) and late-senescence stage (FL5). (B) The sampled flag leaf at five developmental stages. (C) The chlorophyll content. (D,E) The expression level of senescence marker genes SGR and OsNAP. The data are expressed as the mean ± SD of three biological replicates. Statistical significance was defined using Student’s *t*-test: *p* < 0.05 (*) and *p* < 0.01 (**). Figure S2. The KEGG pathway enrichment analysis of DEGs in the five stage-specific modules including MEbrown, MEmagenta, MEturquoise, MEpink and MEblack. The horizontal axis represents the rich factor, while the vertical axis represents the enriched pathway name. The color scale indicates different thresholds of the *p* value, and the size of the dot indicates the number of metabolites corresponding to each pathway. The top 20 pathways ranked by *p*-value are displayed. Figure S3. WGCNA of differential metabolites (DMs). Each row corresponds to a module and each column corresponds to a specific-stage sample. The values in each cell at the row–column intersection represent the correlation coefficients and significance level between modules and traits, the color scale on the right shows correlation values from 1 (red) to −1 (blue), indicating positive to negative correlations. Table S1. The identified 9412 differential expressed genes and functional annotation. Table S2. The genes of identified stage-specific modules by WGCNA. Table S3. GO enrichment analysis of genes in the MEbrown, MEmagenta, MEturquoise, MEpink and MEblack modules, respectively. Table S4. KEGG enrichment analysis of genes in the MEbrown, MEmagenta, MEturquoise, MEpink and MEblack modules, respectively. Table S5. The identified transcription factors in five stage-specific modules. Table S6. The hub genes in five stage-specific modules (MEbrown, MEmagenta, MEturquoise, MEpink and MEblack) based on the connectivity degree. Table S7. The identified 380 differential metabolites (DMs) and functional annotation. Table S8. The identified DMs and classification in five modules by WGCNA. Table S9. The correlation analysis between 40 hub genes and 309 DMs. Table S10. The designed primers used for vector construction and qPCR.
