# Supplementary material for: Time-Course Transcriptome, Metabolome, and Weighted Gene Co-Expression Network Analysis Reveal the Roles of the OsBELH4A Gene in Regulating Leaf Senescence and Grain Yield of Rice
Source: Plants (Basel). 2025 Sep 25;14(19):2973. doi: 10.3390/plants14192973 (PMC12525649; doi:10.3390/plants14192973)
Supplement: Supplementary file 1 [file plants-14-02973-s001.zip › Supplementary Table S8.pdf]

Supplementary Table S8 The identified DMs and classification in five modules by WGCNA.

1. Lipids and lipid-like molecules

class	ID	Metabolite	module
Fatty Acyls	neg_M131T133	Ethylmalonic acid	blue
	neg_M177T78	2-Ethyl-2-Hydroxybutyric acid	blue
	neg_M293T142	Ethyl (S)-3-hydroxybutyrate glucoside	blue
	pos_M319T346	17(18)-Epoxy-5Z,8Z,11Z,14Z-eicosatetraenoic acid	blue
	neg_M277T378	Alpha-Linolenic acid	blue
	pos_M213T264	Dihydrojasmonic acid	blue
	pos_M319T364	17(18)-Epoxy-5Z,8Z,11Z,14Z-eicosatetraenoic acid	green
	neg_M133T52	D-Malic acid	green
	pos_M406T172	Dihydroroseoside	red
	neg_M317T398	5-Hydroxy-6E,8Z,11Z,14Z,17Z-eicosapentaenoic acid	turquoise
	pos_M197T283	Dodeca-2(E),4(E)-dienoic acid	turquoise
	pos_M225T152	13-Oxo-9,11-tridecadienoic acid	turquoise
	pos_M389T172	Dihydroroseoside	turquoise
	neg_M447T144	Methyl 7-epi-12-hydroxyjasmonate glucoside	turquoise
	pos_M232T140	(R)-Butyrylcarnitine	turquoise
	pos_M335T289	Prostaglandin A2	turquoise
	neg_M343T72	Maltitol	turquoise
	neg_M395T261	Prostaglandin D3	turquoise
	pos_M382T126_2	Cis-Zeatin-o-glucoside	turquoise
	pos_M355T362	2-Linoleoylglycerol	turquoise
	pos_M355T406	2-Linoleoylglycerol	turquoise
	neg_M189T140	Adipate semialdehyde	turquoise
	neg_M365T265	12-oxo-10,11-dihydro-20-COOH-LTB4	turquoise
	neg_M365T354	18-Hydroxyeicosatetraenoic acid	turquoise
	pos_M153T53	(2R,4Z)-4-Hepten-2-ol	turquoise
	neg_M365T279	12-oxo-20-dihydroxy-leukotriene B4	turquoise
	neg_M229T211	Dodecanedioic acid	brown
	neg_M686T124_1	3'-Dephosphocoenzyme A	brown
	neg_M225T256	3,4-Methylenesebacic acid	brown
	neg_M271T456	16-Hydroxyhexadecanoic acid	brown
	neg_M293T373	13-HOTE	brown
	neg_M309T332	9-Hydroperoxy-10E,12Z,15Z-octadecatrienoic acid	brown
	pos_M211T236	Jasmonic acid	brown
	pos_M213T370	Cucurbic acid	brown
	pos_M569T278	Leukotriene F4	brown
Glycero lipids	pos_M1040T369	TG 66:21; TG (22:7/22:7/22:7)	blue
	neg_M645T313	MGDG 22:2; MGDG (4:0/18:2)	blue
	neg_M787T432	GlcADG 36:6; GlcADG (18:3/18:3)	blue
	pos_M696T362	Gingerglycolipid B	blue

	pos_M694T340	Gingerglycolipid A	blue
	pos_M474T410	LysoDGTS 16:0	turquoise
	neg_M766T540	GlcADG 34:3; GlcADG (16:0/18:3)	turquoise
	pos_M498T385	LysoDGTS 18:2	turquoise
	neg_M806T442	MGDG 35:6; MGDG (19:2/16:4)	brown
	neg_M790T494	GlcADG 36:5; GlcADG (18:2/18:3)	brown
	neg_M476T367	LysoPE 18:2	blue
	pos_M478T370	LysoPE 18:2	blue
	neg_M503T427	LysoPG 18:4; LysoPG 18:4	blue
	neg_M505T544	LysoPG 18:3; LysoPG 18:3	blue
	neg_M564T366	LysoPC 18:2	blue
	neg_M478T390	LysoPE 18:1	blue
	neg_M562T344	LysoPC 18:3	green
	pos_M518T346	LysoPC 18:3	green
	neg_M540T390	LysoPC 16:0	green
	pos_M496T393	LysoPC 16:0	green
	pos_M454T389	LysoPE 16:0	green
	neg_M452T386	LysoPE 16:0	green
	pos_M476T344	LysoPE 18:3	green
	neg_M474T341	LysoPE 18:3	green
	neg_M554T415	LysoPC 17:0	green
	neg_M466T409	LysoPE 17:0	green
Glycerophospholipids	pos_M520T374	LysoPC 18:2	red
	neg_M520T390	LysoPS 18:2; LysoPS 18:2	turquoise
	neg_M595T544	LysoPI 18:2; LysoPI 18:2	turquoise
	pos_M546T327	PE 22:3; PE (2:0/20:3)	turquoise
	pos_M588T329	PC 22:3; PC (2:0/20:3)	turquoise
	neg_M552T372	LysoPC 17:1	turquoise
	pos_M510T418	1-Heptadecanoyl-sn-glycero-3-phosphocholine	turquoise
	neg_M593T303	LysoPI 18:3; LysoPI 18:3	brown
	neg_M431T432	LysoPA 18:3; LysoPA 18:3	brown
	neg_M526T367	LysoPC 15:0	brown
	pos_M482T370	LysoPC 15:0	brown
	neg_M566T395	LysoPC 18:1	brown
	neg_M872T449	PI 37:4; PI (18:2/19:2)	brown
	pos_M522T398	Plasmenyl-PC 18:0; PC(P-14:0/4:0)	brown
	neg_M438T364	LysoPE 15:0	brown
	neg_M480T433	LysoPE 18:0	brown
	neg_M568T446	LysoPC 18:0	brown
	pos_M524T451	1-Stearoyl-2-hydroxy-sn-glycero-3-phosphocholine	brown
	neg_M381T260	(14S)-14,15-Dihydroxy-8(17),13(16)-labdadien-19-oic acid	blue
Prenol lipids	pos_M301T346	Retinoic acid	blue
	pos_M321T323	Oryzalide B	blue
	neg_M347T296	Gibberellin A112	blue

	pos_M161T283	1-(1-Methylethenyl)-4-(1-methylethyl) benzene	turquoise
	pos_M175T170	1-Methyl-4-(1-methyl-2-propenyl)-benzene	turquoise
	pos_M177T152	(S)-4-(4-Methylphenyl)-2-pentanone	turquoise
	pos_M179T283	2,5-Diisopropylphenol	turquoise
	pos_M209T172	Carvyl propionate	turquoise
	pos_M387T152	Sonchuionoside C	turquoise
	pos_M285T415	13-cis-Retinal	turquoise
	neg_M345T327	Gibberellin A64	turquoise
	pos_M599T337	Idoxanthin	turquoise
	pos_M599T361	Idoxanthin	turquoise
	pos_M599T442	Idoxanthin	turquoise
	neg_M381T250	Cinncassiol A	turquoise
	neg_M599T445	Capsanthin 5,6-epoxide	turquoise
	pos_M317T355	4-Hydroxyretinoic acid	turquoise
	neg_M315T377	Phytocassane E	turquoise
	neg_M313T384	Momilactone A	brown
	neg_M375T248	10'-Apo-beta-carotenal	brown
	neg_M821T420	Licoricesaponin H2	brown
	neg_M315T255	Trilobinone	brown
Sphingo lipids	neg_M690T456	GlcCer[AP] 28:2; GlcCer[AP](t16:1/12:1)	turquoise
	neg_M692T442	GlcCer[AP] 28:1; GlcCer[AP](t16:0/12:1)	turquoise
Sterol Lipids	neg_M331T320	11b-Hydroxyprogesterone	blue
	neg_M363T352	11b,17a,21-Trihydroxypreg-nenolone	blue
	neg_M439T319	Prednisolone phosphate	blue
	pos_M303T376	1-Methylene-5. alpha.-androstan-3. alpha.-ol-17-one	blue
	pos_M303T323	4-Androsten-11.beta.-ol-3,17-dione	blue
	pos_M303T395	1-Methylene-5. alpha.-androstan-3. alpha.-ol-17-one	green
	neg_M439T93	Prednisolone phosphate	turquoise
	pos_M333T335	11b-Hydroxyprogesterone	turquoise
	pos_M275T264	Nandrolone	turquoise
	pos_M303T415	1-Methylene-5.alpha.-androstan-3.alpha.-ol-17-one	turquoise
	neg_M317T287	Estradiol	turquoise
	pos_M335T340	5.beta.-Pregnane-3.alpha.,17-diol-20-one	turquoise
	pos_M275T330	Nandrolone	brown
	pos_M307T443	Oxandrolone	brown
	neg_M331T304	11b-Hydroxyprogesterone	brown
NA	neg_M175T163	2-Isopropylmalic acid	turquoise
	neg_M223T310	Methyl Jasmonic acid	brown

2. Phenylpropanoids and polyketides

class	ID	Metabolite	module
Phenylpropanoic acids	pos_M207T152	Ibuprofen	turquoise
	pos_M207T172	Ibuprofen	turquoise

	pos_M207T313	Ibuprofen	brown
Isoflavonoids	neg_M431T204	Genistin	blue
	neg_M477T191	Genistin	turquoise
	neg_M435T175	4',5,7-Trihydroxy 3,3',6,8-tetramethoxyflavone	blue
	neg_M269T261	Apigenin	blue
	neg_M271T261	Naringenin	blue
	neg_M299T219	Hispidulin	blue
	neg_M299T267	Hispidulin	blue
	neg_M403T245	5,3'-Dihydroxy-6,7,4'-trimethoxyflavone	blue
	neg_M417T192	5,4'-Dihydroxy-3,3'-dimethoxy-6:7-methylenedioxyflavone	blue
	neg_M507T166	Syringetin-3-O-glucoside	blue
	pos_M331T269	3,5-dihydroxy-2-(4-hydroxy-3-methoxyphenyl)-7-methoxy-4H-chromen-4-one	blue
	pos_M301T270	Chrysoeriol	blue
	pos_M347T270	Limocitrin	blue
	pos_M655T196	Malvin	red
	neg_M593T199	Vitexin 4-O-glucoside	red
Flavonoids	pos_M595T201	Vitexin 4-O-glucoside	red
	neg_M623T180	Isoscoparin 2"-O-glucoside	red
	pos_M625T182	Scoparin 2"-glucoside	red
	pos_M627T156	Quercetin-3,4'-O-di-beta-glucoside	turquoise
	neg_M537T207	3',7-Dimethoxy-4',5,8-trihydroxyflavone 8-glucoside	turquoise
	neg_M477T178	6-Methoxyluteolin 3'-glucoside	turquoise
	pos_M317T180	Isorhamnetin	turquoise
	pos_M479T180	Petunidin 3-galactoside	turquoise
	neg_M447T190	Cynaroside	turquoise
	neg_M785T180	Isoorientin 2"-[feruloyl-(->6)-glucoside]	turquoise
	pos_M787T182	Isoorientin 2"-[feruloyl-(->6)-glucoside]	turquoise
	pos_M617T178	Vitexin 4-O-glucoside	turquoise
	pos_M317T244	Isorhamnetin	brown
	neg_M315T241	Isorhamnetin	brown
	neg_M579T183	Cyanidine-3-O-sambubioside	brown
	pos_M161T309	6-Methylcoumarin	turquoise
	neg_M381T244	Foetidin	turquoise
	pos_M207T169	7-Hydroxy-4-(methoxymethyl)coumarin	brown
	neg_M223T168	Sinapic acid	blue
Coumarins and derivatives	pos_M165T162	3-Hydroxycinnamic acid	green
	neg_M385T168	1-O-beta-D-Glucopyranosyl sinapate	red
	pos_M225T169	Sinapic acid	red
	neg_M163T203	3-Coumaric acid	red
	pos_M165T77	3-Hydroxycinnamic acid	turquoise
	neg_M193T163	trans-4-Hydroxy-3-methoxycinnamate	turquoise
	neg_M193T307	3-Hydroxy-4-methoxycinnamic acid	turquoise
	neg_M325T159	2-O-p-Coumaroyl-D-glucose	turquoise

	pos_M225T201	Sinapic acid	turquoise
	pos_M247T285	Heptyl cinnamate	brown
	pos_M195T169	trans-Ferulic acid	brown

3. Lignans, neolignans and related compounds

class	ID	Metabolite	module
Lignan glycosides	pos_M600T166	(7'R)-(+)-Lyoniresinol 9'-glucoside	turquoise
	neg_M537T198	Isolariciresinol 9'-O-alpha-L-arabinofuranoside	brown
NA	neg_M621T447	Thalsimidine	turquoise
	pos_M623T282	Thalsimidine	turquoise

4. Organic acids and derivatives

class	ID	Metabolite	module
Sulfonyls	pos_M95T54	Dimethyl sulfone	turquoise
Organonitrogen compounds	pos_M318T319	Phytosphingosine	turquoise
	pos_M302T337	D-erythro-Sphinganine	turquoise
Organic oxides	neg_M145T241	2-Methylpentanal	turquoise
Alcohols and polyols	neg_M515T165	1,5-Dicaffeoylquinic acid	blue
Carbohydrates and carbohydrate conjugates	neg_M341T138	1-O-Caffeoylglucose	red
Carbonyl compounds	pos_M167T172	Dihydrojasmone	blue
	pos_M127T204	(Z)-5-Octenal	brown
NA	neg_M105T51	Glyceric acid	blue
	pos_M261T55	Mannose 6-phosphate	blue
	neg_M71T53	Malondialdehyde	green
	pos_M184T50	Phosphocholine	red
	pos_M209T155	2,2,6,7-Tetramethylbicyclo[4.3.0]nona-1(9),4-diene-7,8-diol	turquoise
	pos_M368T129	Xanthurenate-8-O-beta-D-glucoside	turquoise
	neg_M289T46	D-Ribose 5-phosphate	turquoise
	neg_M323T83	3-Hydroxy-4-butanolide	turquoise
	neg_M317T85	Arbutin	turquoise
	neg_M271T85	Arbutin	turquoise
	neg_M331T52	4-Glucogallic acid	turquoise
	pos_M139T138	keratan sulfate II (core 2-linked), degradation product 1	turquoise
	pos_M301T139	Salicylic acid beta-D-glucoside	turquoise
	neg_M259T46	D-Glucose 6-phosphate	turquoise
	neg_M353T141	3-Caffeoylquinic acid	turquoise
	neg_M85T74	Diacetyl	turquoise
	pos_M344T160	cis-beta-D-Glucosyl-2-hydroxycinnamate	turquoise
	neg_M195T48	D-Gluconic acid	green

5. Organic acids and derivatives

class	ID	Metabolite	module
Carboxylic acids and derivatives	pos_M150T75	Racemethionine	blue
	pos_M215T122	Val-Pro	blue
	pos_M231T153	Val-Ile	blue
	pos_M233T137	Thr-Leu	blue
	pos_M295T128	Phe-Glu	blue
	pos_M161T52	D-Alanyl-D-alanine	blue
	pos_M186T56	DL-Glutamate	blue
	pos_M147T50	Glutamine	blue
	pos_M132T91_2	Isoleucine	green
	neg_M115T52	Maleic acid	green
	neg_M218T132	D-(+)-Pantothenic acid	red
	neg_M458T125	(6S)-5-Methyltetrahydrofolic acid	turquoise
	neg_M69T73	Propynoic acid	turquoise
	pos_M130T75_2	D-Pipecolinic acid	turquoise
	pos_M130T53	D-Pipecolinic acid	turquoise
	pos_M131T152_2	D-1-Amino-2-pyrrolidinecarboxylic acid	turquoise
	pos_M140T152	DL-Norvaline	turquoise
	neg_M135T49	Peracetic acid	turquoise
	neg_M180T75	L-Tyrosine	turquoise
	pos_M182T77_2	Tyrosine	turquoise
	pos_M182T53	Tyrosine	turquoise
	pos_M237T134	Phe-Ala	turquoise
	pos_M245T172	Leucyl-Leucine	turquoise
	pos_M295T161	Tyr-Leu	turquoise
	pos_M116T51	Proline	turquoise
	neg_M133T136	Propionic acid	turquoise
	pos_M120T51	Threonine	turquoise
	pos_M233T83	Ile-Thr	turquoise
	pos_M260T134	Gln-Leu	turquoise
	pos_M260T79	Leu-Gln	turquoise
	pos_M261T139	Glu-Leu	turquoise
	pos_M265T164	Val-Phe	turquoise
	pos_M302T184	Leu-Gly-Leu	turquoise
	pos_M318T186	Ile-Trp	turquoise
	pos_M427T49	L-Cysteine-glutathione disulfide	turquoise
	neg_M130T89	L-Leucine	turquoise
	pos_M118T54	Betaine	turquoise
	pos_M295T160	Glu-Phe	brown
	pos_M128T121	D-Alanine	brown

	pos_M229T145	Leu-Pro	brown
	pos_M308T53_2	L-Glutathione, reduced	brown
	pos_M308T75_2	L-Glutathione, reduced	brown
Hydroxy acids and derivatives	neg_M169T54	(S)-3-Sulfonatolactate	turquoise
	neg_M261T133	3-hydroxy-3-(3-hydroxyphenyl)propanoic acid-O-sulphate	turquoise
	neg_M149T52_2	D-Lactic acid	turquoise
Keto acids and derivatives	neg_M157T95	2-Oxooctanoic acid	turquoise
Organic phosphoric acids and derivatives	neg_M167T50	Phospho(enol)pyruvic acid	blue
NA	neg_M173T202	cis-Aconitic acid	turquoise
	neg_M117T81	Succinic acid	turquoise
	neg_M173T53_1	trans-Aconitic acid	turquoise
	neg_M191T52	Citric acid	turquoise
	pos_M193T75	Citric acid	turquoise

6. Benzenoids

class	ID	Metabolite	module
Phenols	neg_M93T236_2	Phenol	turquoise
	pos_M185T147	3,4-Dihydroxymandelic acid	turquoise
Benzene and substituted derivatives	neg_M152T92	3-Aminosalicylic acid	blue
	pos_M197T157	3,4-Dimethoxyphenylacetic acid	blue
	neg_M138T136	Aniline	turquoise
	pos_M169T121	2,6-Dihydroxy-4-methylbenzoic acid	turquoise
	neg_M357T261	1,3,5-Triphenylcyclohexane	turquoise
	pos_M189T152	1-(2,3,6-trimethyl phenyl)-3-Buten-2-one	turquoise
	neg_M153T168	Gentisic acid	turquoise
	neg_M217T222	Cinnamyl isovalerate	turquoise
	neg_M365T403	Adipostatin A	turquoise
	pos_M151T122	Phenylglyoxylic acid	turquoise
	pos_M279T122	Sulfamethazine	turquoise
	neg_M137T153	4-Hydroxybenzoic acid	turquoise
	neg_M137T236_2	4-Hydroxybenzoic acid	turquoise
	neg_M163T158	Enol-phenylpyruvate	turquoise
	pos_M139T239	Salicylic acid	turquoise
	pos_M139T155	Salicylic acid	turquoise
	pos_M155T187	2,6-Dihydroxybenzoic acid	turquoise
	pos_M279T184	Dibutyl phthalate	turquoise
	neg_M167T144	Vanillic acid	turquoise
	neg_M209T233	2-Butyl-4-methylphenol	brown
	neg_M243T216	4,4'-Methylenedianiline	brown
Anthracenes	pos_M271T264	Aloe-emodin	blue

NA	neg_M167T129	Homogentisic acid	turquoise

7. Alkaloids and derivatives

class	ID	Metabolite	module
Ibogan-type alkaloids	neg_M703T443	Voacamine	turquoise
	pos_M138T51	Trigonelline	blue
NA	pos_M360T183	Napelline	turquoise
	neg_M715T359	Subsessiline	turquoise

8. Nucleosides, nucleotides, and analogues

class	ID	Metabolite	module
	neg_M346T72	Adenosine monophosphate	blue
Purine	neg_M362T73	Guanosine monophosphate	blue
nucleotides	pos_M364T75	Guanosine 5'-monophosphate	blue
	pos_M348T75	Adenosine 5'-monophosphate	turquoise
	neg_M346T51	Adenosine monophosphate	turquoise
	neg_M565T57	UDP-D-glucose	blue
NA	pos_M348T51	UDP-D-glucose	blue
	neg_M323T51	Uridine 5'-monophosphate	turquoise

9. Organoheterocyclic compounds

class	ID	Metabolite	module
Tetrapyrroles and	neg_M829T190	Uroporphyrin I	turquoise
derivative	pos_M607T458	Phaeophorbide b	turquoise
	neg_M144T154	4-Hydroxyquinoline	turquoise
Quinolines and	neg_M188T154	Kynurenic acid	turquoise
derivatives	pos_M190T156	Kynurenic acid	turquoise
	pos_M206T136	Xanthurenic acid	turquoise
	pos_M124T52	Isonicotinic acid	turquoise
Pyridines and	neg_M152T274	6-Methoxy-pyridine-3-carboxylic acid	turquoise
derivatives	pos_M124T75	Isonicotinic acid	brown
	neg_M455T156	Riboflavin-5'-monophosphate	turquoise
	pos_M86T56	Piperidine	turquoise
Oxepanes	pos_M163T94	Levoglucosan	blue
Oxanes	neg_M349T257	Oryzalic acid A	turquoise
Naphthofurans	neg_M877T276	27-O-demethylrifabutin	brown
	pos_M195T276	Sedanolide	blue
	pos_M195T264	Sedanolide	blue
Lactones	neg_M203T102	3-Hydroxyadipic acid 3,6-lactone	turquoise
	pos_M195T215	Sedanolide	brown
	pos_M195T305	Sedanolide	brown
Indoles and	neg_M219T78	5-Hydroxy-L-tryptophan	blue

derivatives	pos_M205T148	(卤)-Tryptophan	blue
	pos_M162T156	Indole-3-carboxylic acid	turquoise
	neg_M236T129	5-Hydroxyindole-3-acetic acid	turquoise
	pos_M221T98	Indole-3-pyruvic acid	turquoise
	pos_M221T80	Indole-3-pyruvic acid	turquoise
	neg_M203T146	Tryptophan	turquoise
	pos_M177T105	Serotonin	brown
Imidazopyrimidines	pos_M136T79_2	Adenine	turquoise
Heteroaromatic compounds	neg_M155T141	2-Isopropylfuran	turquoise
	neg_M126T74	Pyrrole	brown
	neg_M211T302	2-Heptylfuran	brown
Dihydrofurans	neg_M111T73	2-Furancarboxylic acid	turquoise
	neg_M175T52	Vitamin C	turquoise
	pos_M177T74	Vitamin C	turquoise
	pos_M177T54	Vitamin C	turquoise
Benzopyrans	neg_M365T320	5'-Carboxy-alpha-chromanol	blue
	neg_M319T320	5'-Carboxy-alpha-chromanol	blue
	neg_M319T284	5'-Carboxy-alpha-chromanol	turquoise
	pos_M193T166	Myristicin	turquoise
	pos_M193T309	Myristicin	turquoise
Azoles	neg_M184T139	5-Acetyl-2,4-dimethyloxazole	turquoise
NA	neg_M329T266\neg_M329T218	5,7-dihydroxy-2-(4-hydroxyphenyl)-3,6-dimethoxy-4H-chromen-4-one	blue\turquoise

10. Unknown

ID	Metabolite	module
pos_M171T166	.delta.-Decalactone	blue
pos_M185T75	Chelidonic acid	blue
pos_M293T508	Methyl .gamma.-linolenate	blue
pos_M295T276	9-Oxo-10(E),12(E)-octadecadienoic acid	blue
pos_M353T376	Monolinolenin (9c,12c,15c)	blue
neg_M461T208	Tectoridin	red
pos_M250T162	4-Coumaroylcholine	red
pos_M225T172	Methyl jasmonate	turquoise
neg_M177T156	Coniferyl aldehyde	turquoise
neg_M521T213	Iridin	turquoise
neg_M307T283	3-Cyclopentene-1-octanoic acid, 2-(3-hydroxy-1-penten-1-yl)-5-oxo-	turquoise
neg_M625T154	Herbacetin-3,8-diglucopyranoside	turquoise
pos_M293T264	9-Oxo-10E,12Z,15Z-octadecatrienoic acid	turquoise
pos_M319T332	9-Oxoprosta-10,12Z,14E-trienoic acid	turquoise
pos_M336T341	9-Oxoprosta-10,12Z,14E-trienoic acid	turquoise
pos_M300T322	DErySphingosine	turquoise
neg_M383T226	Artesunate	turquoise
pos_M261T199	Khellin	turquoise

pos_M339T170	4-Methylumbelliferyl .beta.-D-glucopyranoside	turquoise
pos_M593T492	Pheophorbide a	turquoise
neg_M267T49	Ethyl .beta.-D-Glucuronide	turquoise
neg_M435T545	1-Oleoyl-L-.alpha.-lysophosphatidic acid	turquoise
neg_M367T357_2	Methoxyfenozide	brown
pos_M369T360_2	Methoxyfenozide	brown
pos_M133T360	N-Nitroso-N-methyl-3-aminopropionic acid	brown
pos_M291T434	Methyl palmitoleate	brown
neg_M225T46	Allose	brown
neg_M179T49	Allose	brown
neg_M345T267	Luteolin 7-methyl ether	brown
pos_M160T105	2-Methylquinolin-8-ol	brown
pos_M193T246	3-Cyclohexyl-1,1-dimethylurea	brown
pos_M277T376_2	9,12-Octadecadiynoic acid	brown
pos_M293T335	9-Oxo-10E,12Z,15Z-octadecatrienoic acid	brown
pos_M293T388	9-Oxo-10E,12Z,15Z-octadecatrienoic acid	brown
pos_M303T350	Norethandrolone	brown
neg_M293T405	9-Oxo-10(E),12(E)-octadecadienoic acid	brown
pos_M295T409	9-Oxo-10(E),12(E)-octadecadienoic acid	brown
