# Supplementary figures and images for: Time-Course Transcriptome, Metabolome, and Weighted Gene Co-Expression Network Analysis Reveal the Roles of the OsBELH4A Gene in Regulating Leaf Senescence and Grain Yield of Rice
Source: Plants (Basel). 2025 Sep 25;14(19):2973. doi: 10.3390/plants14192973 (PMC12525649; doi:10.3390/plants14192973)

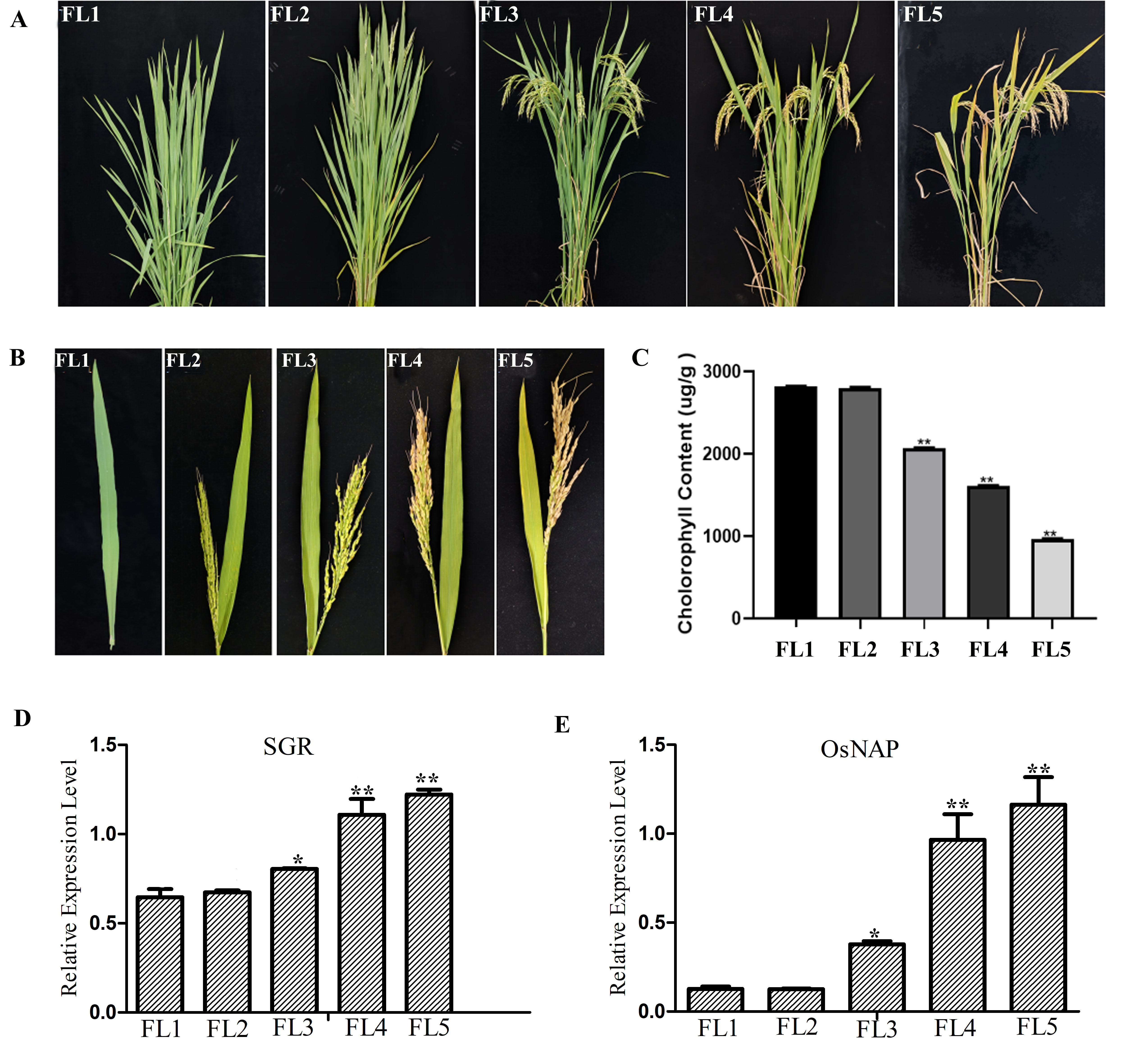

Supplement: Supplementary file 1 [file plants-14-02973-s001.zip › Supplementary Figure S1.jpg]
